# Genome-wide identification of DCL, AGO and RDR gene families and their associated functional regulatory elements analyses in banana (*Musa acuminata*)

**DOI:** 10.1371/journal.pone.0256873

**Published:** 2021-09-02

**Authors:** Fee Faysal Ahmed, Md. Imran Hossen, Md. Abdur Rauf Sarkar, Jesmin Naher Konak, Fatema Tuz Zohra, Md. Shoyeb, Samiran Mondal

**Affiliations:** 1 Faculty of Science, Department of Mathematics, Jashore University of Science and Technology, Jashore, Bangladesh; 2 Faculty of Biological Science and Technology, Department of Genetic Engineering and Biotechnology, Jashore University of Science and Technology, Jashore, Bangladesh; 3 Faculty of Life Science, Department of Biochemistry and Molecular Biology, Mawlana Bhashani Science and Technology University, Tangail, Bangladesh; 4 Faculty of Agriculture, Laboratory of Fruit Science, Saga University, Honjo-machi, Saga, Japan; GC University Faisalabad, PAKISTAN

## Abstract

RNA silencing is mediated through RNA interference (RNAi) pathway gene families, i.e., Dicer-Like (DCL), Argonaute (AGO), and RNA-dependent RNA polymerase (RDR) and their *cis*-acting regulatory elements. The RNAi pathway is also directly connected with the post-transcriptional gene silencing (PTGS) mechanism, and the pathway controls eukaryotic gene regulation during growth, development, and stress response. Nevertheless, genome-wide identification of RNAi pathway gene families such as DCL, AGO, and RDR and their regulatory network analyses related to transcription factors have not been studied in many fruit crop species, including banana (*Musa acuminata*). In this study, we studied *in silico* genome-wide identification and characterization of DCL, AGO, and RDR genes in bananas thoroughly via integrated bioinformatics approaches. A genome-wide analysis identified 3 MaDCL, 13 MaAGO, and 5 MaRDR candidate genes based on multiple sequence alignment and phylogenetic tree related to the RNAi pathway in banana genomes. These genes correspond to the *Arabidopsis thaliana* RNAi silencing genes. The analysis of the conserved domain, motif, and gene structure (exon-intron numbers) for MaDCL, MaAGO, and MaRDR genes showed higher homogeneity within the same gene family. The Gene Ontology (GO) enrichment analysis exhibited that the identified RNAi genes could be involved in RNA silencing and associated metabolic pathways. A number of important transcription factors (TFs), e.g., ERF, Dof, C2H2, TCP, GATA and MIKC_MADS families, were identified by network and sub-network analyses between TFs and candidate RNAi gene families. Furthermore, the *cis*-acting regulatory elements related to light-responsive (LR), stress-responsive (SR), hormone-responsive (HR), and other activities (OT) functions were identified in candidate MaDCL, MaAGO, and MaRDR genes. These genome-wide analyses of these RNAi gene families provide valuable information related to RNA silencing, which would shed light on further characterization of RNAi genes, their regulatory elements, and functional roles, which might be helpful for banana improvement in the breeding program.

## 1 Introduction

Small RNAs (sRNAs) are considered novel riboregulators. These small regulatory RNAs are mainly of two types; microRNAs (miRNAs) and short interfering RNAs (siRNAs), both of which consist of 21–24 nucleotide and are generated from the synthesis processes of double-stranded RNAs (dsRNA) [1,2]. They play key roles in gene silencing by controlling messenger RNA (mRNA) stability, both (pre and post) translation process, or target epigenetic modifications to specific genome positions in plants, animals, and fungi [3]. Vast numbers of sRNA molecules exist in the plant genome. In plants, the regulatory mechanisms of sRNA molecules are well established, and their regulatory processes are controlled by proteins encoded by three RNAi gene families such as DCL, AGO, and RDR and their corresponding regulatory elements [3–9]. In multicellular organisms, DCL, AGO, and RDR genes are the main components of the sRNAs biogenesis process and RNAi pathway, triggering the gene silencing activities [10,11].

The DCL proteins are the major constituents of the sRNAs biogenesis process and have significant roles in post-translational regulation in the RNAi pathway [12–16]. There are six conserved and functional domains for DCL proteins, i.e., DEAD, Helicase-C, DUF1785, PAZ, RNase III, and DSRM, which are the important part of the proteins to be functional [17,18]. AtDCL proteins are precious enzyme that is capable of producing both siRNAs and miRNAs, [19] that are associated with flowering mechanism [20], and development of plant vegetative phase [17,19,21,22]. DCL proteins have structurally and numerically diversified in higher plants, insects, protozoa, and fungi, while a single DCL protein is available in vertebrates [23].

AGO proteins play a crucial and ubiquitous role in the repression of a gene, which are the vital elements of RNAi pathway [16]. AGO proteins have a significant molecular weight (approximately 90–100 kDa) and contain various qualitative functional domains, i.e., Argo-N/Argo-L, DUF 1785, PAZ, ArgoMid, and PIWI [10,24]. An important binding site is present in the PAZ domain. Generally, 5′ end of the phosphodiester bond of the sRNAs can bind to the specific site of the ArgoMid domains of AGO proteins [25], whereas the PIWI domain binds to the 5′ end of the siRNAs [24]. Three groups of AGO protein families are predominant as Ago-like (plants, animals, fungi, and bacteria), PIWI-like (animals), and *C*. *elegens*-specific group, manifest in multicellular and unicellular organisms (i.e., fungi) [24–26]. AGO proteins are mainly associated with RNA silencing mechanisms that play an important role in controlling the transgene-silencing [27], epigenetic silencing mechanisms [28], plant growth [29], and meristem maintenance [30] in different plants species.

The third major RNAi-related protein group is RDR which has a single conserved domain called RdRP [31,32]. The RdRP domain is taken part by the RDR, which creates the RDR proteins as an acting member of the RNA interface gene family [33]. RDR protein family is found in nematodes and fungi [34,35]. However, the regulatory effects of the RDR protein family have not yet been determined in insects and vertebrates [36]. RDR gene families are responsible for various gene silencing mechanisms, including co-suppression, antiviral silencing, chromatin silencing, and PTGS in plants, for example, in Arabidopsis, tobacco, and maize [2,11,37–39]. Moreover, RDR type is essential for antiviral silencing [11,40,41], endogenous gene regulation [42,43], arrangement of heterochromatin and gene resistance [43,44].

Through the advancement of high throughput integrated bioinformatics approaches, several RNAi related DCL, AGO, and RDR gene families have been identified and *in silico* characterized in many economically important plant species such as 28 genes in maize (*Zea mays*) [18] and tomato (*Solanum lycopersicum*) [35], 32 genes in rice (*Oryza sativa*) [45], 22 genes grapevine (*Vitis vinifera*) [46], and pepper (*Capsicum annuum* L.) [47], 20 genes in cucumber (*Cucumis sativus* L.) [48], 51 genes in Brassica (*Brassica* sp.) [49], 23 genes in barley (*Hordeum vulgare*) [50], 36 genes in sugarcane (*Saccharum spontaneum*) [51], 25 genes in sweet orange (*Citrus sinensis*) [5,52] and 38 genes in foxtail millet (*Setaria italica*) [53]. In the model plant species (*A*. *thaliana*), 20 RNAi pathways associated genes such as 4 AtDCL, 10 AtAGO, and 6 AtRDR genes have been identified and characterized [54–57].

Banana (*Musa acuminata*) is a perennial, monocotyledonous major fruit crop grown all over the tropical and sub-tropical country, especially in the African, Asia-Pacific, and Latin American and Caribbean regions [58,59]. Banana contains vitamins (vitamin A, vitamin C, vitamin B6, and vitamin B12), antioxidants, minerals, fiber, starch, sugar and cellulose. Previous studies showed the significant impact of bananas antioxidant on various diseases, for example, anti-hypertension, anti-cancer and anti-diabetes, anti-coronary disease, anti-diarrhea, and defense against infectious disease [60,61]. Thus, various agronomic traits of bananas such as biotic and abiotic stress, disease and pest resistance, and fruit quality are of considerable interest to plant breeders. However, the improvement of bananas through the breeding program has been a great challenge to the breeder for various reasons. Therefore, genetic engineering approaches play a vital role in crop improvement.

So far, characterization and expression analysis of target RNAi pathway genes have comprehensively been conducted in many plant species such as rice [62], soybean [63,64], sugarcane [65], corn [66], strawberry [67], wheat [68], cucumber [48]. However, this approach has limitations in skillful human resources, a well-equipped laboratory, extended time, and experimental budgets. Despite the extensive analysis of RNAi genes using laboratory-based experiments, we can obtain their genome-wide information from many plant species using the knowledge of integrated bioinformatics approaches which might save cost, labor, and longer time demand.

**Fig 1:**
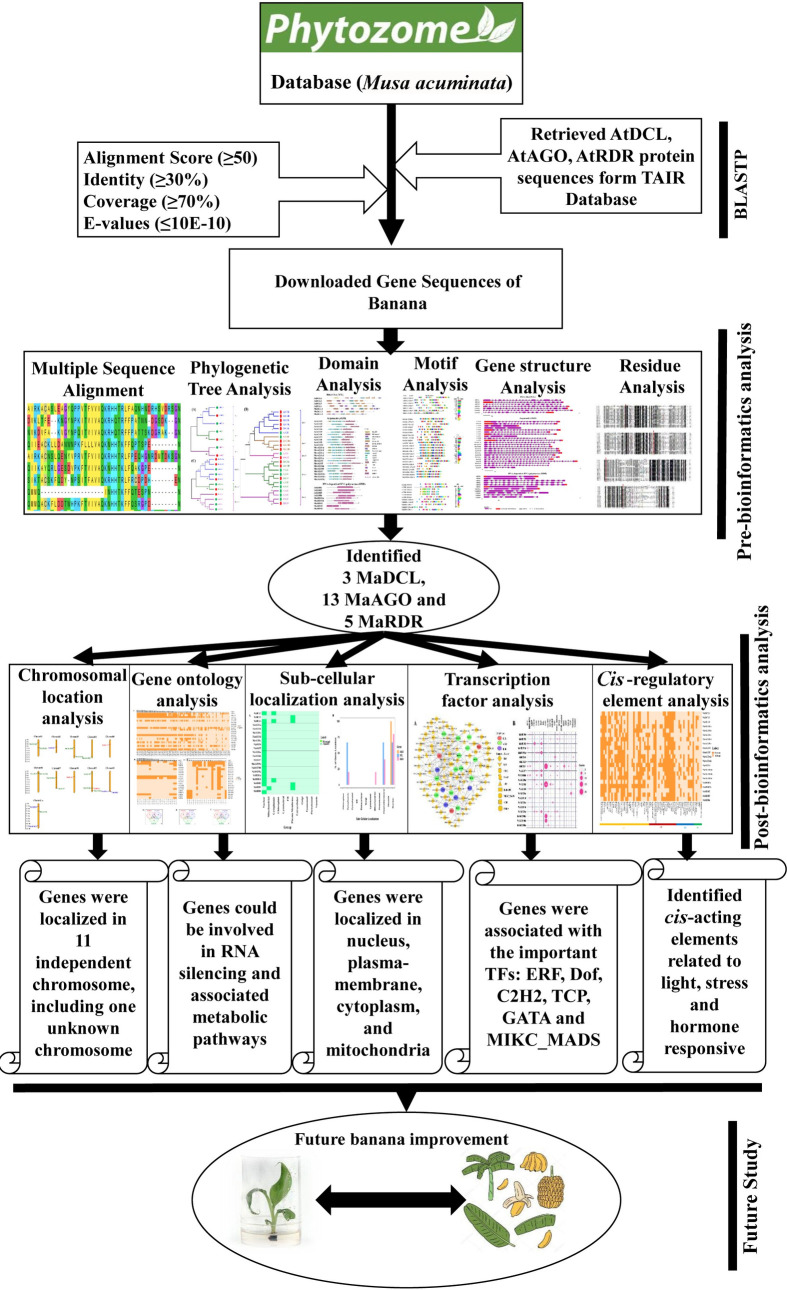
A graphical abstract of our study.

Therefore, in the present study, we performed a comprehensive *in silico* analysis for genome-wide identification of RNAi pathway gene families DCL, AGO, and RDR through bioinformatics approaches such as sequence similarity, phylogenetic relationship, gene structure (domain, motif, and exon-intron numbers), chromosomal localization, GO, sub-cellular localization, regulatory network and sub-network analysis between TFs and candidate genes, prediction of *cis*-acting regulatory elements of TFs in the banana genome. The complete banana genome sequence [69] provides us an excellent opportunity to identify the putative genes related to the RNAi pathway in the entire banana genome, which would be a useful resource in banana improvement programs in the future. We have explained our proposed approach graphically in Fig 1.

## 2 Materials and methods

### 2.1 Genome-wide identification and *in silico* analysis of candidate DCL, AGO and RDR genes in banana

Protein sequences were retrieved from the Phytozome database (https://phytozome.jgi.doe.gov/pz/portal.html) by using the completed genome assembly (v1) of *M*. *acuminata* to identify candidate DCL, AGO, and RDR genes [70]. We further collected the identified sRNA silencing protein sequences of the model plant *A*. *thaliana* (AtDCL1: AT1G01040, AtDCL2: AT3G03300, AtDCL3: AT3G43920, AtDCL4: AT5G20320; AtAGO1: AT1G48410, AtAGO2: AT1G31280, AtAGO3: AT1G31290, AtAGO4: AT2G27040, AtAGO5: AT2G27880, AtAGO6: AT2G32940, AtAGO7: AT1G69440, AtAGO8: AT5G21030, AtAGO9: AT5G21150, AtAGO10: AT5G43810; and AtRDR1: AT1G14790, AtRDR2: AT4G11130, AtRDR3: AT2G19910, AtRDR4: AT2G19920, AtRDR5: AT2G19930, AtRDR6: AT3G49500) from The *Arabidopsis* Information Resource (TAIR) database (http://www.arabidopsis.org) and collected sequences were used to search the protein sequences of *M*. *acuminata*. Furthermore, The Basic Local Alignment Search Tool (BLASTP) program was performed against *M*. *acuminata* in the Phytozome database (Fig 1).

The retrieved candidate protein sequences from *M*. *acuminata* were downloaded with the significant alignment score (≥50) based on BLOSUM62 matrix, identity percentage (≥30%), coverage percentage (≥70%) and the significant E-values (≤10E-10). Only the primary transcript of the sequences was considered to avoid the redundancy of protein sequences in this study. Genomic information, including the primary transcript, genomic length, chromosomal position of a gene, and length of the open reading frame (ORF), protein length, was downloaded from the *M*. *acuminata* genome database deposited in Phytozome. The molecular weight of the identified protein sequences was determined and predicted by using the ExPASyComputepI/Mwtool (https://web.expasy.org/). Identified DCL, AGO, and RDR genes in *M*. *acuminata* genome were named according to the nomenclature based on the phylogenetic of the similar gene family members of the previously named *A*. *thaliana* genes.

### 2.2 Multiple sequence alignments and phylogenetic analysis

The multiple sequence alignments of DCL, AGO, and RDR protein sequences of both *M*. *acuminata* and *A*. *thaliana* were conducted by using the Clustal-W method [71] through the MEGA 11.0 software [72]. Finally, the phylogenetic tree analysis was carried out using the Neighbor-joining method [73] implemented on the aligned sequenced and the 1,000 bootstrap-replicates were used to check this evolutionary relationship. The evolutionary distances were computed using the Equal Input method [74].

### 2.3 Conserved domain and motif analysis

To investigate the conserved domains of DCL, AGO, and RDR gene families in *M*. *acuminata*, protein sequences were retrieved and analyzed using the protein family database (Pfam, https://pfam.xfam.org/) (Fig 1). The maximum number of significant functional conserved domains of *M*. *acuminata* (MaDCL, MaAGO, and MaRDR) that are similar to the *A*. *thaliana* AtDCL, AtAGO, and AtRDR proteins were selected.

We used the Multiple Expectation Maximization for Motif Elicitation (MEME) webserver to investigate the conserved motifs in all of the predicted DCL, AGO, and RDR proteins (http://meme.sdsc.edu/meme4_3_0/cgi-bin/meme.cgi) [75]. This analysis was performed using the following parameters (i) an optimum motif width as ≥6 and ≤50; (ii) a maximum number of motif of 20. Any Motifs that did not match the structural domains in each protein family were rejected.

### 2.4 Gene structure and chromosomal localization analysis

The gene structure (domain structure, exon-intron organization) of predicted genes were analyzed using the online program Gene Structure Display Server (GSDS2.0: https://gsds.cbi.pku.edu.cn) [76]. Furthermore, to compare the exon-intron structure of the predicted genes in *M*. *acuminata*, the selected gene structures were compared with the *A*. *thaliana* gene structure. Chromosomal location of predicted MaDCL, MaAGO, and MaRDR genes was mapped using the online tool MapGene2Chromosome V2 (http://mg2c.iask.in/mg2c_v2.0/) and considered the physical position of the genes in different scaffold locations.

### 2.5 Gene ontology and sub-cellular localization analysis

To determine the relationship of predicted RNAi-related genes with the different clusters of molecular pathways, the GO analysis was performed using the online tool of Plant Transcription Factor Database (PlantTFDB, http://planttfdb.cbi.pku.edu.cn//) [77]. We determined the corresponding *p*-values by the Fishers test and Benjamini-Hochberg’s correction. The *p*-value <0.05 was considered to be a statistically significant level for GO enrichment results of the respective predicted genes. We predicted sub-cellular location of identified MaDCL, MaAGO, and MaRDR proteins into the various organelles of the cell using their protein sequences by a powerful and high accuracy web server Plant Sub-cellular Localization Integrative Predictor (PSI) [78].

### 2.6 Regulatory network analysis between TFs and RNAi related genes in *M*. *acuminata*

To study the regulatory relationship and network analysis between TFs and RNAi-related genes in *M*. *acuminata*, we extensively studied the PlantTFDB (http://planttfdb.cbi.pku.edu.cn//) [77]. Initially, we identified the TFs, which were closely associated with RNAi-related genes in *M*. *acuminata*. Then, constructed a regulatory network and visualized the network using regulatory network visualization tool Cytoscape 3.7.1 [79].

### 2.7 Promoter *cis*-acting regulatory elements analysis

To investigate the promoter *cis*-acting regulatory elements of MaDCL, MaAGO, and MaRDR gene families, upstream region (1.5 kb genomic sequences) of the start codon (ATG) of each RNAi gene were retrieved. Then, we analyzed the stress-response associated promoter *cis*-acting regulatory elements through online prediction analysis using Signal Scan search program from the Plant CARE database (http://bioinformatics.psb.ugent.be/webtools/plantcare/html/) [80]. We classified the analyzed promoter *cis*-acting regulatory elements into five groups; LR, SR, HR, OT, and unknown function. The known function and reported promoter *cis*-acting regulatory elements of MaDCL, MaAGO, and MaRDR are shown separately.

## 3 Results and discussion

### 3.1 *In silico* identification of DCL, AGO, and RDR genes in banana genome

To identify banana RNA silencing genes, AtDCL, AtAGO, and AtRDR retrieved protein sequences were used as query sequences to construct a Hidden Markov Model (HMM) (Fig 1). We identified three genes encoding DCL proteins (MaDCLs), 13 thirteen genes encoding AGO proteins (MaAGOs), and five genes encoding RDR proteins (MaRDRs) in the banana genome database based on the HMM profile analysis. The identified RNA silencing genes, their chromosomal location, and structural features (ORF length, gene length and intron number), protein profile (molecular weight of the encoded protein, isoelectric point (pI) are presented in Table 1. All conserved domains; DEAD, Helicase-C, Dicer-dimer, PAZ, RNase III, and DSRM were predicted in the polypeptide sequence of the three MaDCL loci which confirmed the identity of plant DCL family. The identified MaDCLs ORF ranged from 14058bp to 15156bp, corresponding to MaDCL1 (GSMUA_Achr8T12350_001) and MaDCL3 (GSMUA_Achr5T10880_001) with potentially encoded amino acids (aa) 1818 and 1598 aa (Table 1). According to the pI values of the MaDCLs proteins, MaDCL1 and MaDCl2 showed the acidic properties, whereas only MaDCl3 demonstrated the highest pI value 8.19 which corresponds to the basic properties.

**Table 1:** Basic information of *M*. *acuminata* DCL, AGO and RDR gene families.

Serial number	Gene name	Accession Number	Chromosomal location	ORF (bp)	Gene Length (bp)	No. of Intron	Protein		
							Molecular Weight (kD)	Protein Length (aa)	pI
MaDCL									
1	MaDCL1	GSMUA_Achr8T12350_001	chr8:9122581–9136638	5457	14058	23	205.57	1818	6.27
2	MaDCL3	GSMUA_Achr5T10880_001	chr5:7850534–7865689	4797	15156	28	180.68	1598	8.19
3	MaDCL4	GSMUA_Achr9T22460_001	chr9:27543800–27558358	4092	14559	18	154.51	1363	5.86
MaAGO									
1	MaAGO1a	GSMUA_Achr1T17950_001	chr1:13367712–13375758	3168	8047	23	117.80	1055	9.33
2	MaAGO1b	GSMUA_Achr3T13970_001	chr3:13109171–13117273	3183	8103	23	117.77	1060	9.46
3	MaAGO1c	GSMUA_Achr3T27070_001	chr3:26673724–26683909	3231	10186	24	120.41	1076	9.42
4	MaAGO1d	GSMUA_Achr1T07120_001	chr1:5633224–5641557	3174	8334	21	118.31	1057	9.38
5	MaAGO1e	GSMUA_AchrUn_randomT09260_001	chrUn_random:43037280–43048055	3195	10776	23	119.73	1064	9.32
6	MaAGO4	GSMUA_Achr9T05150_001	chr9:3336565–3342357	2748	5793	21	102.69	915	9.07
7	MaAGO5	GSMUA_Achr6T04510_001	chr6:3088655–3104476	3714	15822	29	137.55	1237	8.81
8	MaAGO6a	GSMUA_Achr9T22630_001	chr9:27732300–27739327	2691	7028	21	101.18	896	9.06
9	MaAGO6b	GSMUA_Achr9T27810_001	chr9:31996792–32001180	2616	4389	21	98.06	871	9.25
10	MaAGO7	GSMUA_Achr7T26400_001	chr7:27702683–27705523	1914	2841	7	73.00	637	9.00
11	MaAGO10a	GSMUA_Achr7T13220_001	chr7:10612033–10619409	2841	7377	23	107.10	946	9.07
12	MaAGO10b	GSMUA_Achr4T30720_001	chr4:28191159–28198319	2802	7161	21	105.76	933	9.30
13	MaAGO10c	GSMUA_Achr6T05310_001	chr6:3572705–3579999	2787	7295	21	104.90	928	9.15
MaRDR									
1	MaRDR1a	GSMUA_Achr10T29960_001	chr10:32256776–32264398	3201	7623	5	121.98	1066	7.94
2	MaRDR1b	GSMUA_AchrUn_randomT26010_001	chrUn_random:127790235–127793988	2895	3754	4	109.61	964	5.99
3	MaRDR2	GSMUA_Achr10T31260_001	chr10:33450046–33460104	3285	10059	9	124.39	1094	6.53
4	MaRDR5	GSMUA_AchrUn_randomT03480_001	chrUn_random:17098685–17104861	1407	6177	8	54.11	468	7.52
5	MaRDR6	GSMUA_Achr2T08230_001	chr2:12303509–12309120	3072	5612	6	116.28	1023	8.47

On the other hand, identified all 13 MaAGO genes exhibited an N-terminal PAZ and a C-terminal PIWI conserved domain in their polypeptide sequences which are the major components of the plant AGO protein family. The identified MaAGOs ORF ranged from 2841 to 15822 bp, which corresponded to MaAGO7 (GSMUA_Achr7T26400_001) and MaAGO5 (GSMUA_Achr6T04510_001) with the potential encoded 637 and 1237 aa (Table 1). The genomic length of the identified MaAGO genes varied from 2841 bp to 15822 bp, which are produced by the MaAGO genes, MaAGO7 (GSMUA_Achr7T26400_001) and MaAGO5 (GSMUA_Achr6T04510_001), respectively. The coding potentiality of MaAGO7 and MaAGO5 are 637 aa and 1237 aa, respectively. The pI values of the predicted all MaAGOs proteins showed higher basic properties (pI value 8.81~9.46).

Our HMM analysis predicted the RdRP conserved domain in MaRDR gene family. The genomic length of the five identified MaRDRs varied from 3754 bp to 13305 bp, which are corresponding with MaRDR1b (GSMUA_AchrUn_randomT26010_001) encoded protein length 964 aa and 460 aa, respectively. The pI values of the MaRDRs proteins demonstrate that the proteins are more likely to be basic, where only the MaRDR6 has the highest pI value of 8.47, which showed the basic properties.

The pI values are widely distributed (1.99–13.96) in various plant species [81]. Also, pI values of plant proteins are greatly involved in post-translational modifications and biochemical functions in plant RNAi gene family [82].

### 3.2 Multiple sequence alignment of DCL, AGO and RDR proteins in banana and *Arabidopsis*

**Fig 2:**
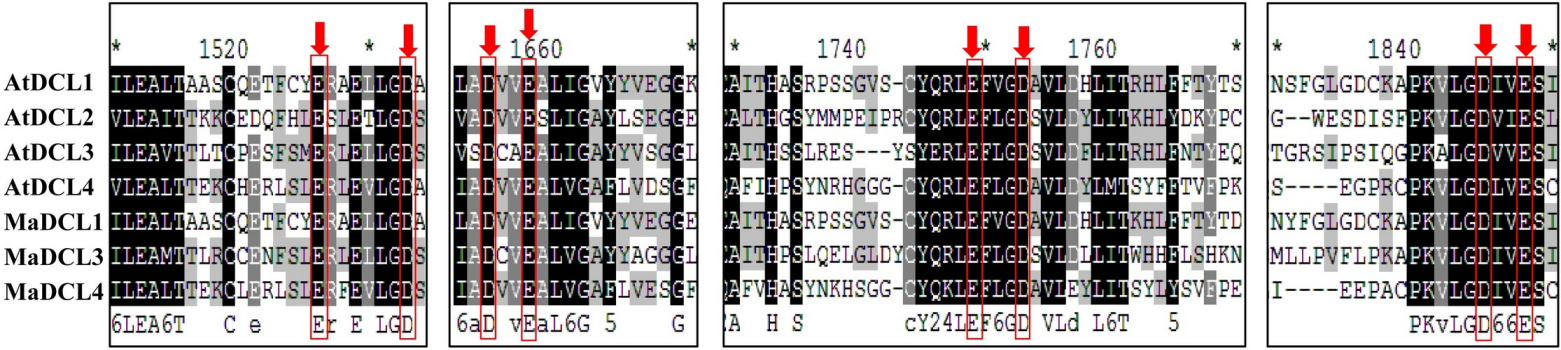
The multiple sequence alignment analysis of RNase III domains (RIBOc I and II) of the aa sequences of *M*. *acuminata* and *Arabidopsis* DCL proteins by Clustal-W program in MEGA 11. The downward red arrows indicate the position of conserved The two RNase III domains at the glutamate (E), aspartate (D), glutamate (D), aspartate (E) (EDDE) position.

**Fig 3:**
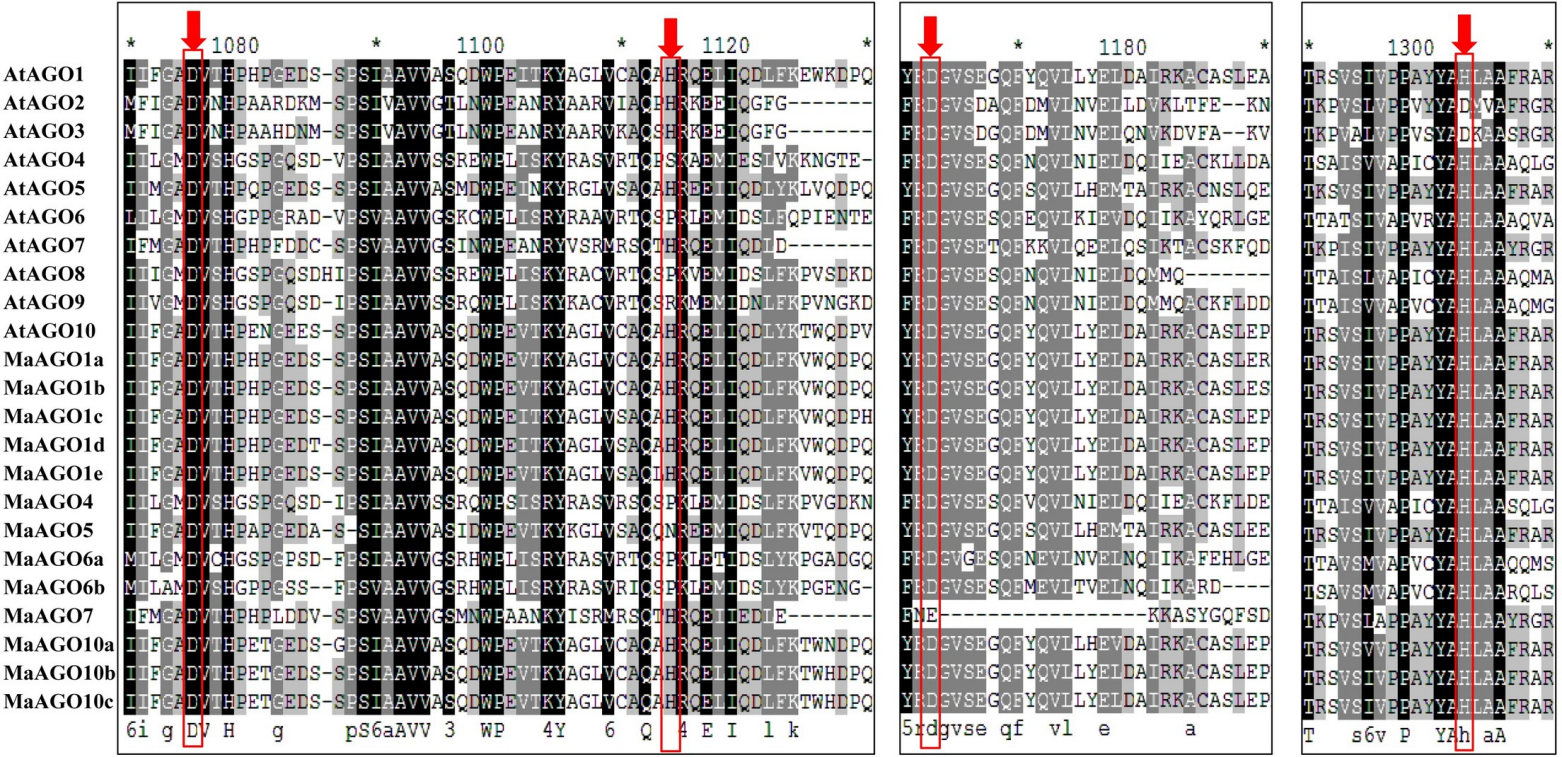
PIWI domain of the aa sequences of *M*. *acuminata* and *Arabidopsis* AGO proteins by Clustal-W program in MEGA 11. The downward red arrows indicatethe conserved DDH triad of PIWI domain and the conserved H798 positions.

**Fig 4:**
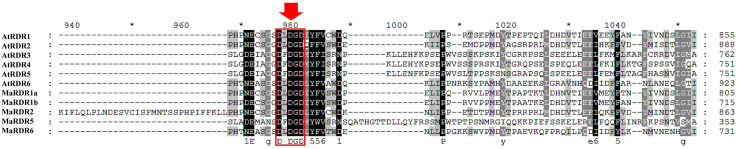
RdRP conserved domain of the aa sequences of *M*. *acuminata* and *Arabidopsis* RDR proteins by Clustal-W program in MEGA 11. The conserved DxDGD catalytic motif are surrounded by the red box.

We obtained the multiple sequence alignment by aligning the predicted MaDCL, MaAGO, and MaRDR protein sequences to the reference sequence of AtDCL, AtAGO and AtRDR (Figs 2–4). The alignment results revealed that the RNase III catalytic sites of the predicted MaDCL proteins in the two RNase III domains at the glutamate (E), aspartate (D), aspartate (D), glutamate (E) (EDDE) position with the orthologs of AtDCLs except for AtDCL3 where aspartate (D) replaced by glutamine (Q) (Fig 2). The three metal-chelating conserved catalytic residues (D = aspartate, D = aspartate, and H = histidine) are found in the PIWI domain, which was first identified in AtAGO1 [45] and responsible for endonuclease activity [83,84]. Further, the alignment of 10 AtAGOs and 13 MaAGOs proteins demonstrated the conserved DDH triad residues of PIWI domains of both AGOs (Fig 3). Moreover, sequence alignment of AtRDRs with the predicted MaRDRs proteins exhibited the DxDGD catalytic motif of the RdRP conserved domain (Fig 4).

The DDH/H motif was detected in the predicted proteins of MaAGO1a, MaAGO1b, MaAGO1c, MaAGO1d, MaAGO1e, MaAGO10a, MaAGO10b, and MaAGO10c, which are similar to AtAGO1 and AtAGO10 proteins (Table 2). The DDH/P motif was found in MaAGO4, MaAGO6a, and MaAGO6b proteins, which are similar to AtAGO6 proteins but dissimilar to AtAGO4 proteins (DDH/S) (Table 2). The DDH/N motif was only observed in MaAGO5 proteins, which are dissimilar to AtAGO5 proteins (DDH/H) (Table 2). Another motif DEH/H was predicted in MaAGO7 proteins, whereas the DDH/H motif was identified in AtAGO7 proteins (Table 2). In this study, MaAGO4 and MaAGO5 proteins catalytic residues histidine (H) at 786^th^ position were replaced to proline (P) and asparagines (N), which are replaced by the fourth serine (S) and histidine (H) residue at 798^th^ position (Table 2). Additionally, MaAGO7 represented one replaced PIWI domain catalytic residues in the second glutamate (E) at 786^th^ position was replaced by the aspartate (D) residue at 895^th^ position (Table 2). Motif analysis results revealed that DDH catalytic residues structure of PIWI domains is not fully conserved in all MaAGOs proteins in bananas.

**Table 2:** Comparison of the AGO proteins in PIWI domains between *M*. *acuminata* and *A*. *thaliana*.

Serial No	*M*. *acuminata v1*		*A*. *thaliana*^*b*^	
	AGO	Motif^a^	AGO	Motif^a^
1	MaAGO1a	DDH/H	AtAGO1	DDH/H
2	MaAGO1b	DDH/H	AtAGO4	DDH/S
3	MaAGO1c	DDH/H	AtAGO5	DDH/H
4	MaAGO1d	DDH/H	AtAGO6	DDH/P
5	MaAGO1e	DDH/H	AtAGO7	DDH/H
6	MaAGO4	DDH/P	AtAGO10	DDH/H
7	MaAGO5	DDH/N		
8	MaAGO6a	DDH/P		
9	MaAGO6b	DDH/P		
10	MaAGO7	DEH/H		
11	MaAGO10a	DDH/H		
12	MaAGO10b	DDH/H		
13	MaAGO10c	DDH/H		

^a^Comparison of conserved motif corresponds to D760, D845, H986/H798 of *Arabidopsis* AGO1; D: aspartate, H: histidine, P: proline, R: arginine, S: serine and Y: tyrosine.

^b^From [45].

In *Arabidopsis*, the PIWI domain of AGO proteins contains DDH/H, DDH/S, and DDH/P motif, which are necessary for their *in vitro* endonuclease activity [83,85,86]. Replacement in the DDH/H conserved motif of PIWI domains in the identified MaAGOs proteins based on the motif analysis due to natural mutation or genetic diversity in the MaAGOs populations. Replacement of aa residues in MaAGOs proteins may reflect reduced endonuclease activity, or replaced aa residues may play significant gene functions in bananas. We can confirm the gene function changes by introducing the reporter genes together with the MaAGO genes and observing their expression analysis in transient expression assay using model plant species such as *Nicotiana*, *Arabidopsis*. So, further gene function analysis is required to understand the proper functions of the PIWI domain with replacements in catalytic residues of MaAGO proteins.

### 3.3 Phylogenetic relationship of DCL, AGO and RDR proteins in banana and *Arabidopsis*

**Fig 5:**
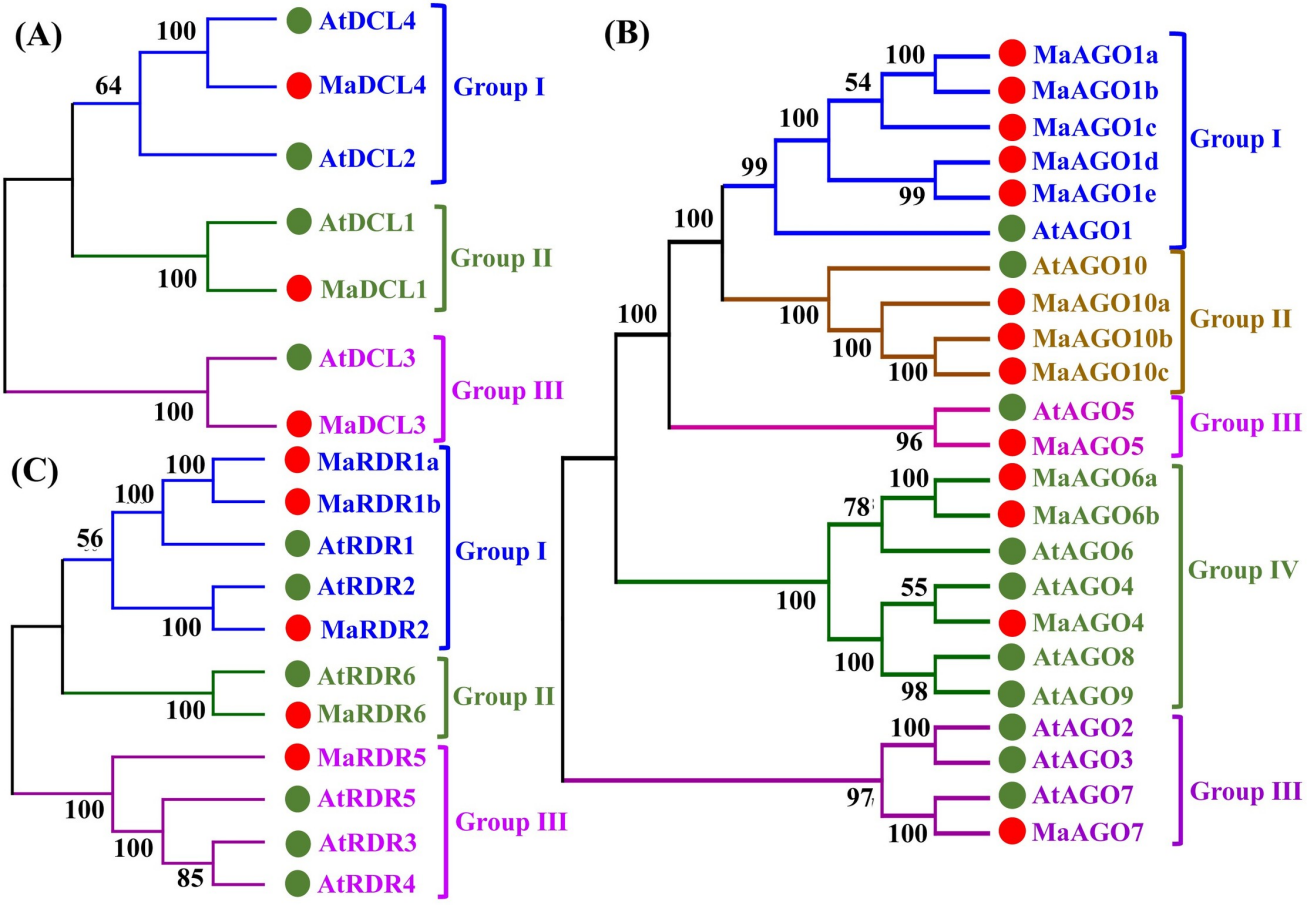
Phylogenetic tree for (A) DCL proteins (B) AGO proteins and (C) RDR proteins from *M*. *acuminata* and *Arabidopsis*. All the phylogenetic trees were constructed using the neighbor-joining method and the numbers at the nodes indicate the percentages of bootstrap values from 1000 replications. The accession number and the abbreviations of proteins from *M*. *acuminata* are tabulated in (Table 1) while *A*. *thaliana* are given in section 2.1. In phylogenetic tree, different groups are represented by different colors; red circles are mentioned genes of *M*. *acuminata* and green circles are mentioned genes of *A*. *thaliana*.

We determined the evolutionary relationship between DCL, AGO and RDR proteins of banana and *Arabidopsis* using each full-length protein sequence by phylogenetic tree analysis (S1–S3 Files and Fig 5A–5C). The phylogenetic tree analysis results revealed that three MaDCL proteins (MaDCL1, MaDCL3 and MaDCL4) were clustered into three Groups (Group I-III) along with their corresponding DCL proteins in *Arabidopsis* with well-supported bootstrap values (S1 File and Fig 5A). The MaDCL1 and MaDCL3 proteins were closely clustered with AtDCL1 and AtDCL3 in Group II and Group III, respectively. The MaDCL1 and MaDCL3 comprised proteins are DCL1 and DCL3 subfamily on the basis of higher sequence similarity with the AtDCL1 and AtDCL3, respectively. The MaDCL4 gene is clustered with AtDCL4 and AtDCL2, which is included in Group I. However, it is noted that MaDCL4 is DCL4 subfamily based on higher sequence similarity with the AtDCL4 gene. The members of the DCLs play key roles in sRNA biogenesis process and are involved in leading the long dsRNAs into mature sRNAs [12,14]. Specifically, based on AtDCL1 protein functions, we can assume that MaDCL1 may be involved in development, environmental stress condition and flowering mechanism [1,43,44,87]. According to AtDCL2 and AtDCL3 functions, we can expect that MaDCL3 will be regenerating the siRNAs and trans-acting small interfering RNA (ta-siRNAs) participate in vegetative phase development, disease resistance and flowering mechanism [87,88]. We can implicit based on the AtDCL4 function that MaDCL4 will be related with ta-siRNA metabolism and acts on RNA-dependent methylation (RdDM)-mediated epigenetic maintenance during post-transcriptional silencing [22,89].

According to the phylogenetic tree analysis, AGO genes of flowering plant species are mainly divided into three clusters; Cluster 1 (AGO1/5/10), Cluster 2 (AGO2/3/7), and Cluster 3 (AGO4/6/8/9), which is similar to our study [90]. We identified 13 AGO genes from *M*. *acuminata* that were classified into five Groups (Group I-V) (S2 File and Fig 5B). In group I, five banana proteins named MaAGO1a, MaAGO1b, MaAGO1c, MaAGO1d, and MaAGOe were clustered with AtAGO1. The five banana proteins are AGO1 subfamily on the basis of higher sequence similarity with the AtAGO1. Group II comprises of three banana proteins (MaAGO10a, MaAGO10b and MaAGO10c) together with AtAGO10 proteins from *Arabidopsis*. The three banana proteins are similar to AGO10 subfamily according to higher sequence similarity with the AtAGO10. The MaAGO5 gene is clustered with AtAGO5, which is included in Group III and it is AGO5 subfamily according to higher sequence similarity with the AtAGO5. Group IV includes three genes from banana (MaAGO4, MaAGO6a and MaAGO6b) and four genes from *Arabidopsis* (AtAGO6, AtAGO4, AtAGO8 and AtAGO9), but MaAGO4 is AGO4 subfamily on the basis of higher sequence similarity with the *A*. *thaliana* AGO protein AtAGO4. Moreover, the rest of the two banana genes (MaAGO6a and MaAGO6b) are included AGO6 subfamily on the basis of higher sequence similarity with the AtAGO6. Among Group V genes, MaAGO7 genes cluster with AtAGO2, AtAGO3, and AtAGO7 genes, but MaAGO7 exists in AGO7 subfamily on the basis of higher sequence similarity with the AtAGO7. *Arabidopsis* genome encodes 10 AGO proteins which have been involved in the RNA silencing mechanism [27,91]. According to AtAGO1 role, we can assume that MaAGO1 may be linked with miRNA and transgene-silencing mechanism [27,92]. According to AtAGO4 function, we can anticipate that MaAGO4 may be involved with endogenous siRNAs activity and required for epigenetic silencing [28,93]. Based on AtGAO7 and AtAGO10, we can expect that MaAGO7 and MaAGO10 may be required for the conversion plant adult phase from the juvenile stage [29] and development of meristem tissue [30,94].

The phylogenetic tree analyses also revealed four groups of RDR genes (Group I-III) (S3 File and Fig 5C). The RDR genes obtained from bananas were designated as MaRDR1a, MaRDR1b, MaRDR2, MaRDR5, and MaRDR6. Group I includes three genes from banana (MaRDR1a, MaRDR1b and MaRDR2) and two genes from *Arabidopsis* (AtRDR1 and AtRDR2), but MaRDR1a and MaRDR1b is RDR1 subfamily on the basis of higher sequence similarity with the *A*. *thaliana* AGO protein AtRDR1. In addition, the MaRDR2 protein exists in the RDR2 subfamily based on the sequence similarity with the *A*. *thaliana* AGO protein AtRDR2. Similarly, the MaRDR6 protein is grouped (Group II) with the AtRDR6 protein and contained RDR6 subfamily according to the sequence similarity with AtRDR6. In Group III, the MaRDR5 protein is clustered with AtRDR3, AtRDR4, and AtRDR5 genes but closely clustered with AtRDR5. The MaRDR5 is included in the RDR5 subfamily on the basis of higher sequence similarity with a single *Arabidopsis* protein AtRDR5. RDRs proteins can regenerate dsRNAs from ssRNA to initiate a signal for RNA silencing mechanism [95,96]. Based on the AtRDR1 function, we can implicit that MaRDR1 could be promoted by a viral infection or salicylic acid and major components for RNA silencing pathway, antiviral defense, and transgenes silencing in many plants species [38,57,97,98]. In relation to AtRDR2 function, we can assume that MaRDR2 may be involved in generating siRNA and associated with chromatin modification [21,99]. According to the AtRDR6 function, we can expect that the MaRDR6 could produce the ta-siRNA precursor and serve in antiviral defense by degradation of RNA molecule [100].

In our analysis, MaDCL2, MaAGO2, MaAGO3, MaAGO8, MaAGO9, MaRDR3 and MaRDR4 were found to be absent in the whole banana genome. These results indicate their functional diversity which would be helpful for further banana improvement.

### 3.4 Conserved domain and motif analysis of DCL, AGO and RDR proteins in banana and *Arabidopsis*

**Fig 6:**
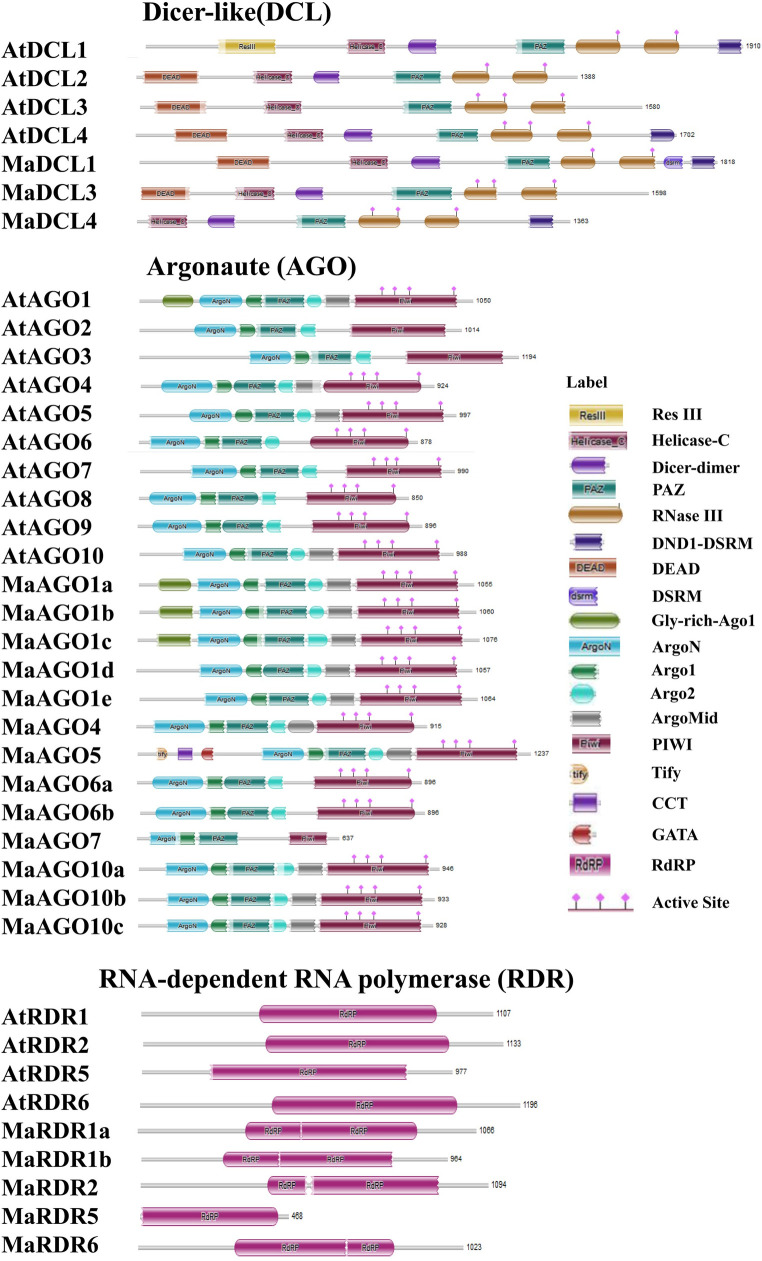
The conserved domains of the predicted MaDCL, MaAGO, and MaRDR proteins were drawn by Pfam database information. Where, Helicase conserved C-terminal domain: Helicase-C; Dicer dimerization domain: Dicer-dimer; PAZ domain: PAZ; Ribonuclease III domain: RNase III; double-strand RNA binding domain from DEAD END PROTEIN 1: DND1-DSRM; DEAD/DEAH box helicase domain: DEAD; Double-stranded RNA binding motif: DSRM; Glycine-rich region of Argonaut: Gly-rich_Ago1; N-terminal domain of argonaute: ArgoN; Argonaute linker 1 domain: ArgoL1; Argonaute linker 2 domain: ArgoL2; Mid domain of argonaute: ArgoMid; PIWI domain: PIWI; Tify domain: Tify; CCT motif: CCT; GATA zinc finger: GATA; RNA dependent RNA polymerase: RdRP; Type III restriction enzyme domain: Res III.

The domain analysis results showed that most functional domains are well conserved in the DCL, AGO, and RDR families from banana and *Arabidopsis* (Fig 6). The MaDCL proteins demonstrated all significant conserved domains; DEAD/Res III, Helicase-C, Dicer-dimer, PAZ, RNase III, and DSRM (Fig 6). The previous studies revealed that these predicted domains play crucial roles in protein activity in plants [22,101,102]. Previously it has been found that the combined activity of two DCL genes has played a crucial role in plant defense against viral infection [103]. The DCLs proteins are responsible for the cleavage of dsRNAs into 21–24 nucleotide long sRNAs. The PAZ domain of DCL proteins mainly functions to bind siRNA and dsRNA, which is cleaved by the two RNase III catalytic functional domains. The endonuclease enzyme-containing RNA-induced silencing complex (RISC) is given by these sRNAs, which encourage the AGO proteins to debase the target homologous RNAs with the arrangement integral to the sRNAs [43,45]. These are also engaged with gene silencing at a transcriptional level by executing chromatin reorganization [21,104].

AGO proteins are mainly characterized by two domains; an N-terminal PAZ, and a C-terminal PIWI domain [10,24,105–107]. Both PAZ and PIWI domains are predicted in all the MaAGO proteins. The predicted AGOs functional domains demonstrated the similarity with the previously reported AtAGO proteins [84]. Previous studies have reported that the PAZ and PIWI domain of AGOs plays a critical role in RNase activity [24,108,109]. Both domains had identical homology with RNase H, which binds to the 5’ end of the siRNA of the target RNA and cleaves the target RNA, thereby demonstrating that the sRNAs are complementary sequences [25,85]. The predicted PAZ and PIWI conserved domains of MaAGO proteins which might play important functional role in synthesizing the double-stranded RNA into single-stranded RNA and stimulate the target RNA degradation process [25,108,110]. The Gly-rich Ago1 domain predicted in MaAGO1a, MaAGO1b, and MaAGO1c proteins is similar to AtAGO1. The Gly-rich Ago1 domain coordinates the binding with the ribosome to enhance AGO protein stimulation for the RNA silencing process [111]. Beside these, the Tify, CCT, and GATA domains are found only in the MaAGO5 protein. The Tify domain is a highly specific conserved domain in plants. Binding with the CCT and GATA, Tify domain is defined as a novel TFs that regulate various developmental processes and respond to biotic and abiotic stresses in plants [112–114]. MaAGO5 protein could possibly perform a major role related to various stresses in bananas which will be clarified by further characterization of this protein in detail.

RDRs are involved in starting a new RNAi silencing process by synthesizing dsRNAs using a single-stranded RNAs (ssRNAs) as templates. A single conserved domain RdRP is present in RDR proteins which possesses a catalytic β’ subunit of RdRP motif [33,45,115–117]. We predicted the typical RdRP domain in all MaRDR proteins in our analysis, which are showed the similarity with RdRP conserved domain of AtRDRs.

**Fig 7:**
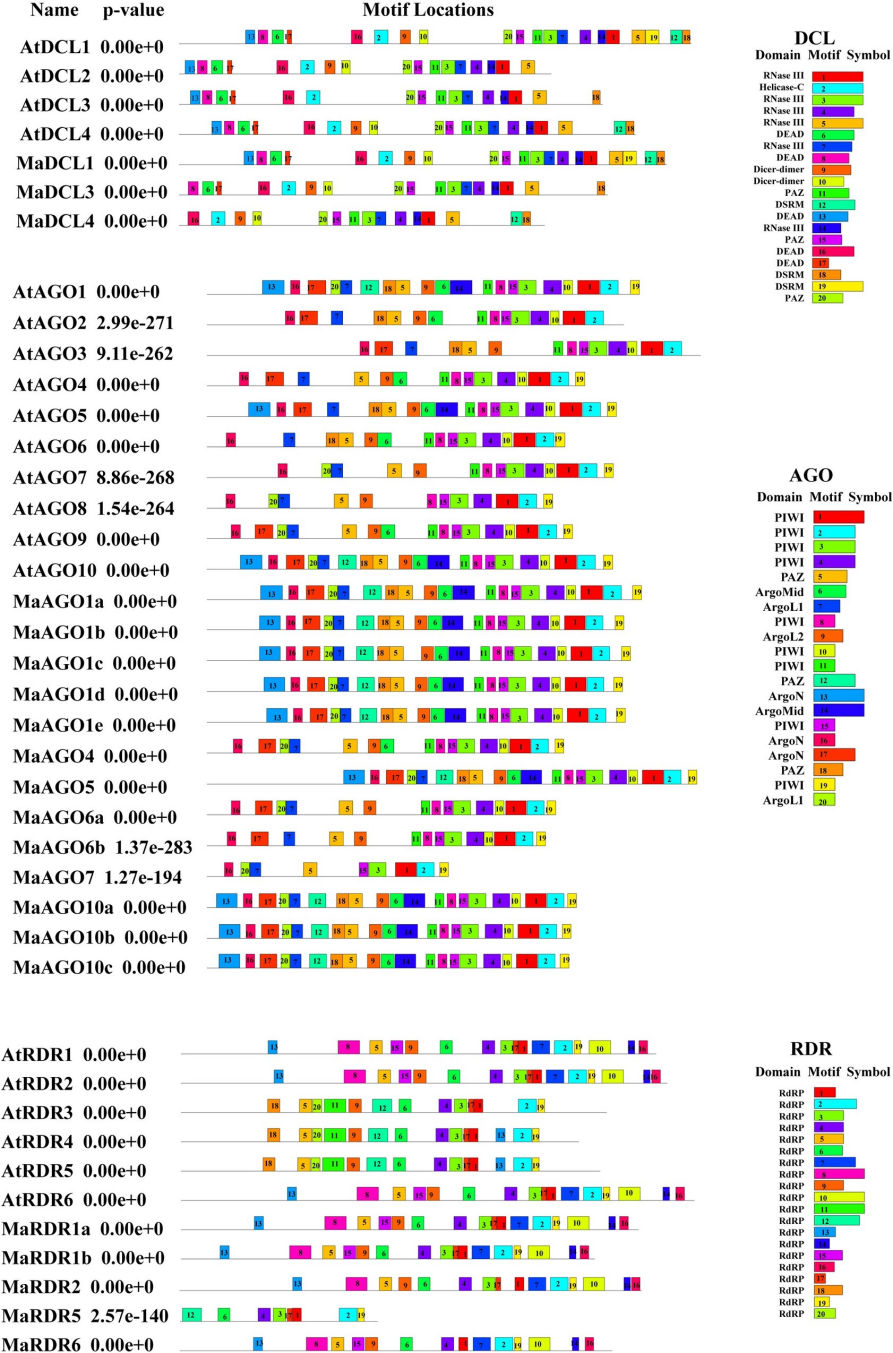
The conserved motifs of the predicted MaDCL, MaAGO and MaRDR protein families are drawn using MEME-suite (a maximum of 20 motifs are displayed). Each color represents different motifs in the predicted proteins domains.

Prediction of motifs insight into a protein sequence has the critical clues to further characterize their functional regulatory roles for gene expression [75]. We predicted typical well-distributed motifs and conserved them in MaDCL, MaAGO, and MaRDR proteins (Fig 7).

We observed a maximum of 20 motifs in both proteins of MaDCL and AtDCL. Possibly, MaDCL1 will appear highly functional as like AtDCL1. However, MaDCL1 comprises of 20 motifs that are similar to the paralogs AtDCL1. The MaDCL3 and MaDCL4 contained 17 and 15 motifs, whereas 16 and 19 motifs were found in AtDCL3 and AtDCL4 proteins, respectively. We were not found motif 13 of DEAD domain in MaDCL3. Moreover, motif 6, 8, 13, and 17 of DEAD domain in MaDCL4 were absence as compared with the AtDCL4 protein. The absence of DEAD motifs in both MaDCL3 and MaDCL4 will possibly show a functional diversity between Arabidopsis and banana.

On the other hand, we also predicted maximum of 20 motifs in MaAGOs. We found all 20 motifs in MaAGO1 (MaAGO1a, MaAGO1b, MaAGO1c, MaAGO1d, and MaAGO1e), and MaAGO10 (MaAGO10a, MaAGO10b, and MaAGO10c), which exhibited higher conservation with their paralogs AtAGO1 and AtAGO10s. These results reflect that the MaAGO1 and MaAGO10 proteins are highly homologous and will show functional similarities like AtAGO1 and AtAGO10s. However, some motif variability was observed in AGO4, AGO5, AGO6a, AGO6b, an AGO7 between the *Arabidopsis* and banana. The motif 20 of ArgoL1 domain was not detected in AtAGO4 but present in MaAGO4. The motif 12 of PAZ domain also was not found in AtAGO5, whereas present in the MaAGO5. However, motif 20 of ArgoL1 domain of in MaAGO5 was absent in this analysis. The motif 17 of ArgoN domain was not detected in MaAGO6a, and MaAGO6b. Another motif 20 of ArgoL1 domain was absent in AtAGO6, whereas this motif was present in the MaAGO6a only. Importantly, motif 18 of PAZ domain was not found in AtAGO6 but were present in MaAGO6a and MaAGO6b in our analysis. The presence of conserved catalytic PAZ domain could lead to enhance the function of target RNA processing by endonucleolytic cleavage in these AGO proteins [46]. Out of two motifs (6, and 14) of ArgoMID domain, only motif 6 was detected in AtAGO6. Although, both 6, and 14 motifs of AgroMID domain were not found in both MaAGO6a, and MaAGO6b. The motif 9 of ArgoL2 domain was completely absent in MaAGO7 protein. The motif variability in MaAGOs compared with AtAGOs suggests the diverse functional roles in RNAi silencing mechanism. We predicted 8–16 numbers of RdRP conserve motif in the MaRDR family. Among the MaRDR family, MaRDR1a and MaRDR1b showed the motif similarity with their paralogs AtRDR1. These results indicate their close functional similarity, which need to be investigated in detail by wet-lab characterization in the future. Moreover, other MaRDRs proteins, MaRDR2, MaRDR5 and MaRDR6 proteins contained 15, 8 and 14 conserved motifs, respectively, which showed discrepancy with their paralogs AtRDR2, AtRDR5 and AtRDR6. The motif 15 of RdRP domain was absent in MaRDR2 proteins. However, maximum numbers of motif (5, 9, 11, 13, 18, and 20) of RdRP domain was not detected in MaRDR5 as compared with their paralogs AtRDR5 proteins. Motifs 3, and 17 of RdRP was not observed in MaRDR6 protein. The analysis of conserved domains and motif showed that the majority of the conserved domains and motifs were well conserved in MaDCL, MaAGO, and MaRDR proteins. The variation of conserved domains and motifs distribution of all these three proteins indicated their possible diverse functional roles.

### 3.5 Analysis of gene structure and chromosomal location of DCL, AGO and RDR proteins in banana and *Arabidopsis*

**Fig 8:**
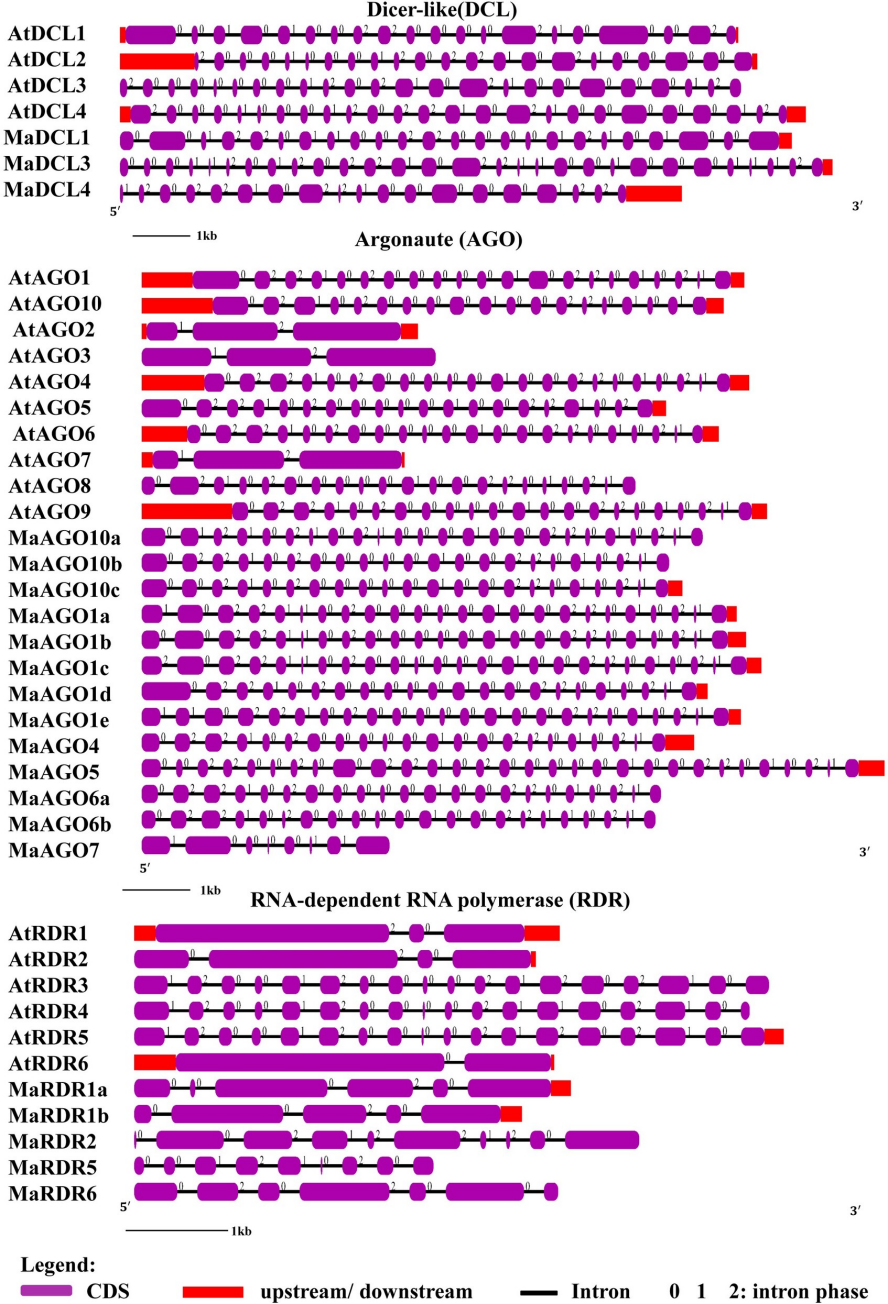
Gene formation of the predicted MaDCL, MaAGO, and MaRDR proteins in *M*. *acuminata* with *Arabidopsis* by using Gene Structure Display Server (GSDS 2.0, http://gsds.cbi.pku.edu.cn/index.php) [76].

The predicted MaDCL, MaAGO, and MaRDR genes showed well-conserved gene structure having the similarity with the reference *Arabidopsis* genes according to the gene structure analysis (Fig 8). The exon-intron numbers of predicted MaDCLs displayed higher numbers compared to the AtDCLs except for MaDCL4 (Fig 8 and Table 1). The MaDCLs intron numbers [12–17,21,23–25,104] demonstrated similarity with AtDCLs. Out of 13 MaAGO genes, 12 MaAGO genes exhibited 22–29 intron, except for MaAGO7 that has only 8 introns (Fig 8). The MaAGOs showed maximum variable numbers of intron (8–30), which were closely similar to the gene structures of AtAGOs. On the other hand, five MaRDR genes displayed 5–10 numbers of the intron in their gene structure, which are shown similarity with AtRDR gene structure except for MaRDR5 (Fig 8). A higher similarity of MaDCL, MaAGO, and MaRDR gene structures with their orthologs *Arabidopsis* suggesting their closely similar functional roles in RNAi pathway.

**Fig 9:**
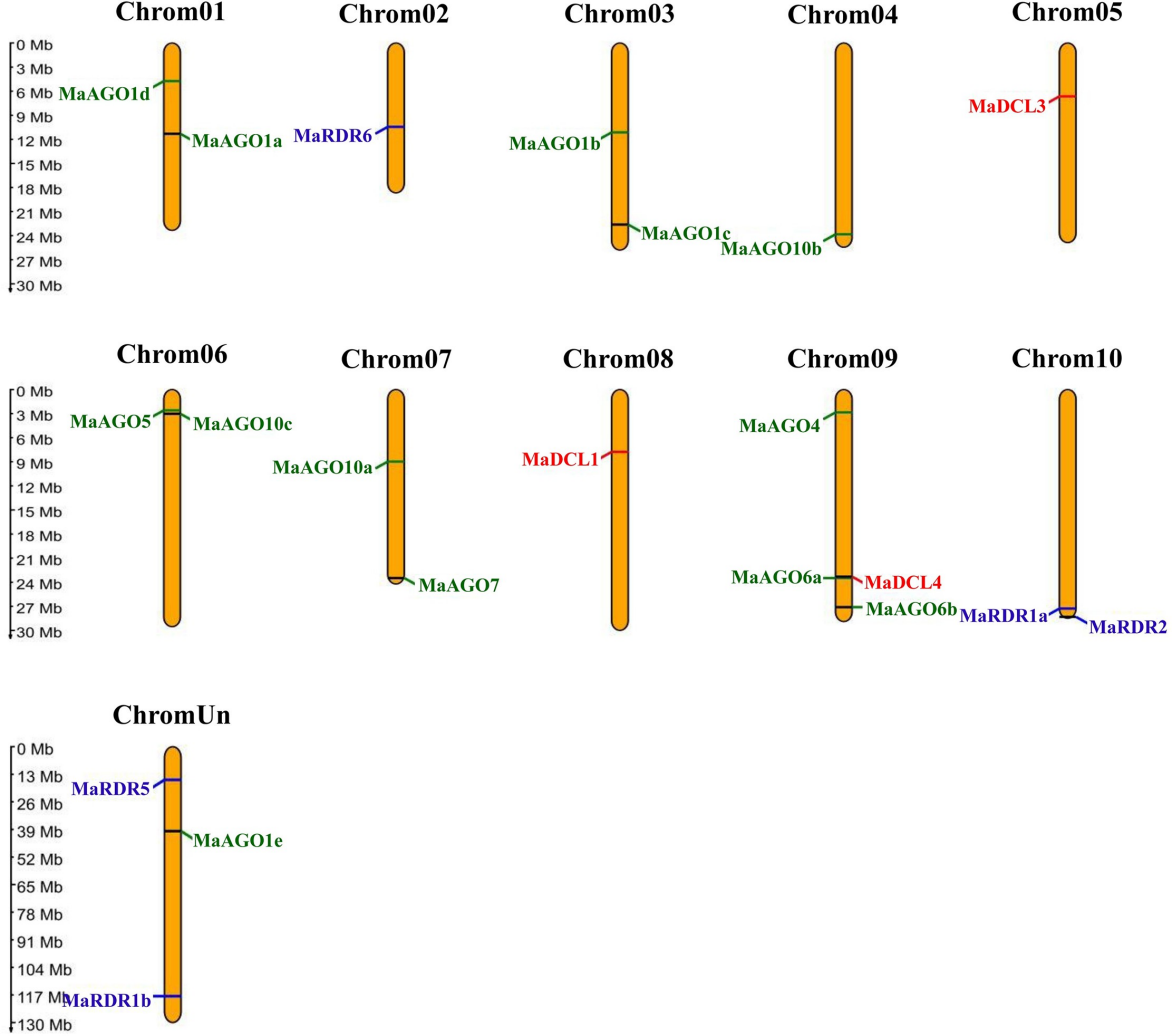
The genomic location of the predicted MaDCL, MaAGO, and MaRDR genes. The scale to indicate the chromosomal length is provided on the left. The ChrUn means the unknown chromosome.

The chromosomal localization analysis results demonstrated that mapped MaDCL, MaAGO, and MaRDR genes were localized in 21 different scaffolds across the 11 independent chromosomes, including one unknown chromosome of the banana’s entire genome (Fig 9, Table 1). The MaDCLs, MaAGOs, and MaRDRs showed a unique scaffold position and were distributed throughout the 11 chromosomes of the banana genome.

The three MaDCL genes, MaDCL1, MaDCL3, and MaDCL4, were located in the three independent chromosomes, i.e., chromosome 5 (MaDCL3), chromosome 8 (MaDCL1), and chromosome 9 (MaDCL4). MaAGO genes are positioned in the chromosome 1 (MaAGO1a, MaAGO1d), chromosome 3 (MaAGO1b, MaAGO1c), chromosome 4 (MaAGO10b), chromosome 6 (MaAGO5, MaAGO10c), chromosome 7 (MaAGO7, MaAGO10a), chromosome 9 (MaAGO4, MaAGO6a, MaAGO6b) and unknown chromosome (MaAGO1c). Besides this, MaRDR genes appear in chromosome 2 (MaRDR6), chromosome 10 (MaRDR1a, MaRDR2), and the unknown chromosome (MaRDR5). Gene pairs (MaAGO5, MaAGO10c) and (MaRDR1a, MaRDR2) were closely located to each other in the genomic location of chromosomes 6 and 10, respectively. These results suggest that these pairs of genes could be expressed in a diverse pattern due to their close genomic location, and further study can be performed under various stress conditions.

### 3.6 Analysis of gene ontology of DCL, AGO and RDR proteins in banana

**Fig 10:**
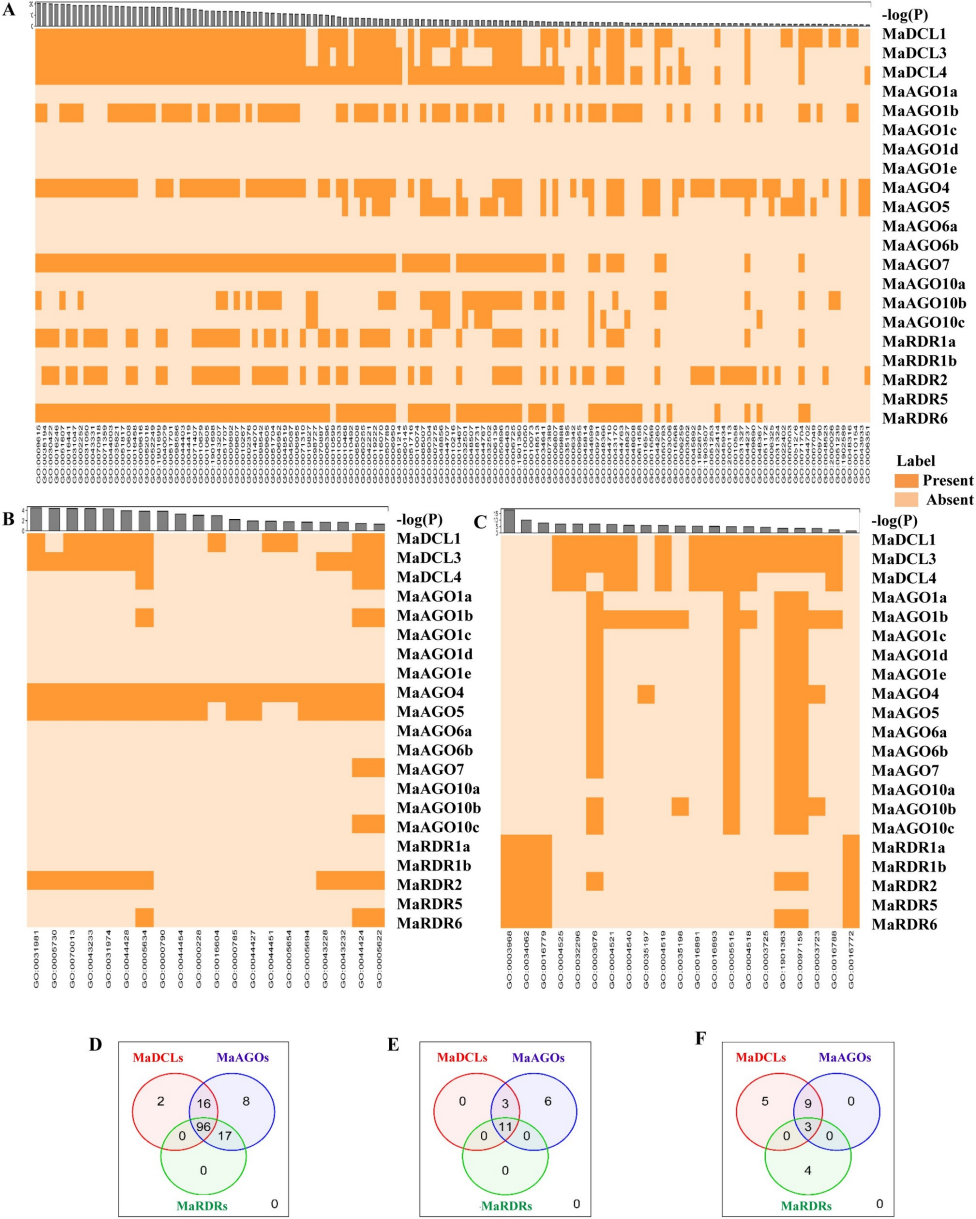
The Heatmap for the predicted GO terms corresponding to the predicted RNAi genes is represented for (A) biological process (B) cellular components and their association to the genes whether they are present or absent. The *p*-value corresponds to the GO terms are shown in the histogram adjacent to the Heatmap using−*log*_10_(*p−value*). The Ven diagrams are drawn to investigate the shared GO terms by MaDCL, MaAGO, and MaRDR gene families considering the (C) biological process (D) cellular components (E) molecular functions.

We hypothesized the biological functions of the predicted RNAi-related genes in detail through GO analysis (Fig 10 and S4 File). The GO analysis results indicated that 9 genes (MaDCL1, MaDCL3, MaDCL4, MaAGO1b, MaAGO4, MaAGO7, MaRDR1a, MaRDR2, and MaRDR6) took part in gene silencing functions (GO: 0016458; p-value: 5.60E-18) and PTGS pathway (GO: 0016441; *p*-value: 3.20E-20). Previous studies suggested that RNAi affects gene silencing through the PTGS mechanism in plants [118]. A detailed analysis of predicted genes revealed the relationship between RNAi and bananas, which would be involved in the cleavage of mRNA in bananas. Based on the GO analysis results, four RNAi genes (MaDCL1, MaDCL3, MaDCL4, and MaAGO1b) exhibited the endonuclease activity among the predicted 16 RNAi associated genes (GO: 0004519; *p*-value: 1.70E-06) (S4 File) in the cells. These GO analysis results demonstrated a link with RISC mediated degradation. The RISC mediates the protein degradation of a cell. Previous studies suggest that AGO (RNAi protein) has an important role in the degradation of protein, which is termed as endonucleolytic cell activity. This endonucleolytic activity of the cells results in the PTGS for mRNA substrate [119]. We identified 16 genes (MaDCL1, MaDCL3, MaAGO1a, MaAGO1b, MaAGO1c, MaAGO1d, MaAGO1e, MaAGO4, MaAGO5, MaAGO7, MaAGO6a, MaAGO6b, MaAGO10a, MaAGO10b, MaAGO10c, and MaRDR2) related to nucleic acid binding activity (GO: 0003676; *p*-value: 1.20E-07). We also identified 5 genes (MaDCL1, MaDCL3, MaAGO1b, MaAGO4, and MaAGO10b) associated with RNA binding activity (GO: 0003723; *p*-value: 4.00E-04). Moreover, we identified 16 genes (MaDCL1, MaDCL3, MaDCL4, MaAGO1a, MaAGO1b, MaAGO1c, MaAGO1d, MaAGO1e, MaAGO4, MaAGO5, MaAGO6a, MaAGO6b, MaAGO7, MaAGO10a, MaAGO10b, and MaAGO10c) which were linked with protein binding activities (GO: 0005515; *p*-value: 9.30E-06). These results indicate that RNAi proteins are involved in the conducted RISC and RNAi mechanism. Our predicted MaAGO proteins exhibited PAZ and PIWI domains, which are greatly responsible for their endonucleases activity [83,85,86]. GO analysis revealed that predicted genes are involved in various important biological functions. The predicted 11 genes (MaDCL1, MaDCL3, MaDCL4, MaAGO1b, MaAGO4, MaAGO5, MaAGO7, MaAGO10b, MaRDR1a, MaRDR2, and MaRDR6) are involved in biological regulation activities (GO:0050789; *p*-value: 6.00E-07), 9 genes (MaDCL1, MaDCL3, MaDCL4, MaAGO1b, MaAGO4, MaAGO7, MaRDR1a, MaRDR2, and MaRDR6) are related to negative regulation of gene expression (GO:0010629; *p*-value:3.60E-15). However, 8 genes (MaDCL1, MaDCL3, MaDCL4, MaAGO4, MaAGO7, MaRDR1a, MaRDR2, and MaRDR6) are associated with fragmentation of dsRNA (GO: 0031050; *p*-value: 3.00E-19), and 5 genes (MaDCL1, MaDCL3, MaDCL4, MaAGO7, and MaRDR6) are predicted to be produced of ta-siRNAs involved in RNAi (GO: 0010267; *p*-value: 7.90E-14). Among the predicted candidate RNAi associated banana genes, 9 genes (MaDCL1, MaDCL3, MaDCL4, MaAGO1b, MaAGO4, MaAGO7, MaAGO10b, MaRDR1a, and MaRDR6) are responsible for responding to the virus (GO:0009615; *p*-value:1.60E-21). We drew the Ven Diagram to observe the shared GO terms for three clusters of the RNAi-associated gene families considering biological process, cellular components, and molecular functions (Fig 10). The Ven Diagram analysis results showed that many MaDCL, MaAGO, and MaRDR genes are common in the GO pathway. According to our study, MaDCL, MaAGO, and MaRDR genes are appeared to be involved in 96 biological processes (Fig 10D). We also observed that predicted RNAi-related genes are shown 11 and 3 common groups of GO pathways in the case of cellular components and molecular functions in bananas, respectively (Fig 10E and 10F). These results suggested that large numbers of RNAi genes are associated with different biological functions, cellular components, and molecular functions in bananas.

### 3.7 The sub-cellular localization of the predicted proteins in banana

**Fig 11:**
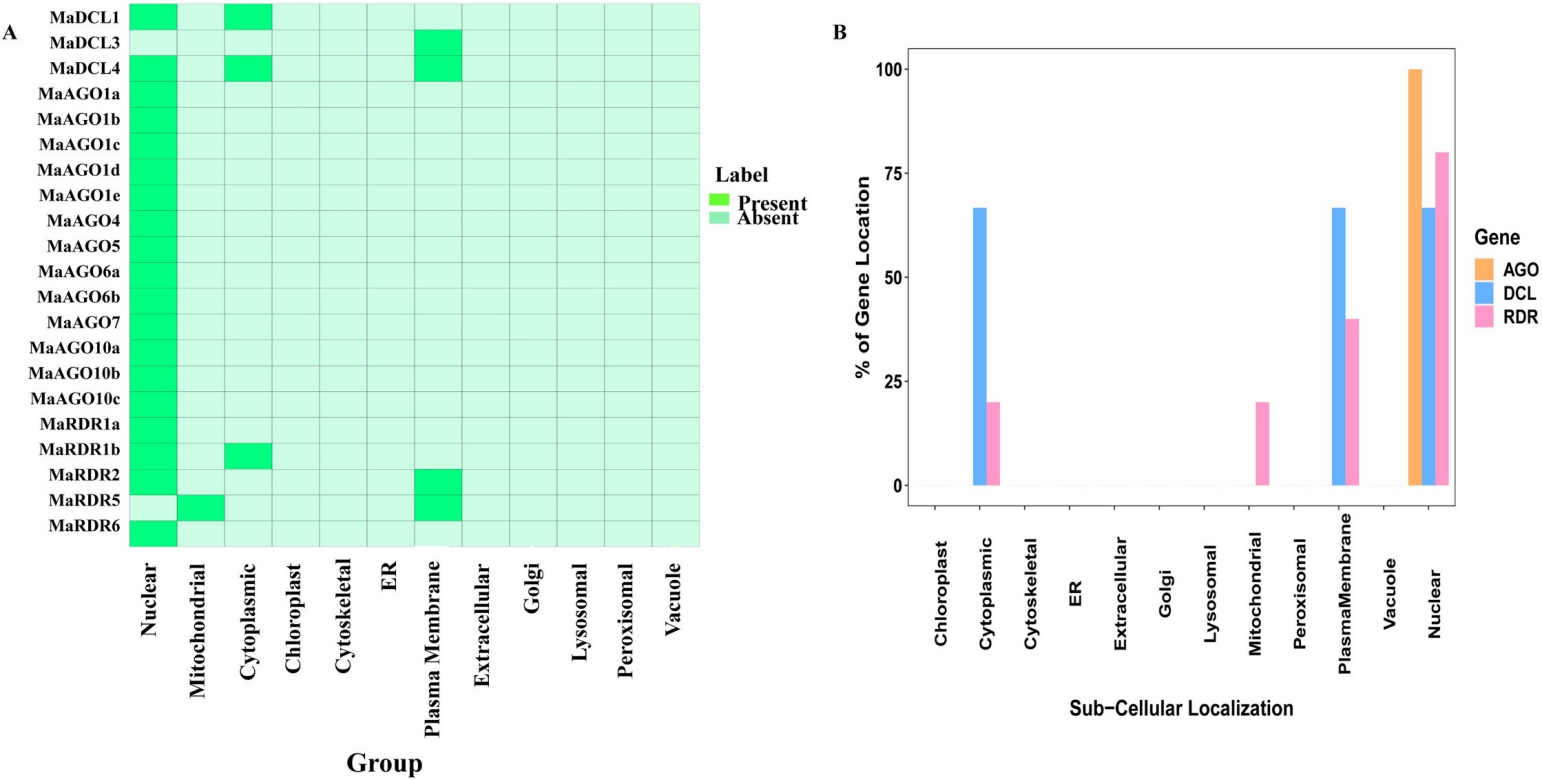
Sub-cellular localization analysis for the (A) MaDCL, MaAGO, and MaRDR proteins. (B) The percentage of protein appeared in different cellular organelles. In this study, predicted proteins were analyzed in Nuclear, Mitochondrial, Cytoplasmic, Chloroplast, Cytoskeletal, Endoplasmic reticulum (ER), Plasma Membrane, Extracellular, Golgi, Lysosomal, Peroxisomal, and Vacuole.

The biological processes of a eukaryotic cell are linked with the sub-cellular localization of specific proteins. The cellular location of protein helps us to understand their functional roles at the cellular level [120,121]. Sub-cellular localization analysis revealed that identified MaDCL, MaAGO, and MaRDR proteins were localized only in the nucleus, plasmamembrane, cytoplasm, and mitochondria (Fig 11). It was observed that MaDCL1 protein is distributed in the nucleus and cytoplasmic region. However, MaDCL3 proteins appeared only in plasma-membrane. On the other hand, MaDCL4 proteins occur in the nucleus, cytoplasmic region, and plasma membrane. Surprisingly, all MaAGO proteins are predicted to be localized only in the nucleus. Among the 5 MaRDR proteins MaRDR1a and MaRDR6 are present in the nucleus only. MaRDR1b protein is predicted in both nucleus and cytoplasm organelles. The MaRDR2 protein is distributed in the nucleus and plasma membrane. Besides this, MaRDR5 protein is abundant in mitochondria and plasma membranes. Based on protein sequences, a computational method for sub-cellular localization of DCL, AGO, and RDR proteins has been conducted in *C*. *sinensis* and predicted them in cell organelles, such as nucleus, cytoplasm, plasma membrane, mitochondria, and plastid [5]. The PTGS and transcriptional gene silencing process occur in the cytoplasm of a cell for targeted mRNA degradation [122,123]. The RNAi proteins are directly involved in RISC-mediated cleavage activities in PTGS process by DCL, AGO, and RDR proteins [119]. The occurrences of PTGS in the cytoplasmic region indicate that candidate protein dominantly participates in this process [122,123]. Moreover, previous studies revealed that Arabidopsis RNAi proteins AGO4 and DCL3 localized in the nucleus and coordinately leading the RNAi silencing process [124]. Our computational-based prediction provides important clues associated with the functions of identified DCL, AGO, and RDR proteins in the RNAi pathway.

### 3.8 Regulatory relationship between transcription factors and RNA interference genes in banana

TFs play a key role in various biological processes in living organisms, in particular, in plants. The plant TFs are involved in regulating diverse functions, e.g., responses to biotic and abiotic stresses, growth, development, metabolism, and defense against microbial infection [125–129]. In plants, TFs act as a molecular switch or key regulators of several functional genes that are expressed under particular stress, growth, and developmental conditions. There are various TFs such as MYB, CBF/DREB1, HSF, AP2/EREBP, Dof, ERF, NAC, MIKC_MADS, WRKY, bZIP ERF, TGA6, and BOS1 families that exist in plants and functions under various stresses and developmental conditions [128–134].

**Fig 12:**
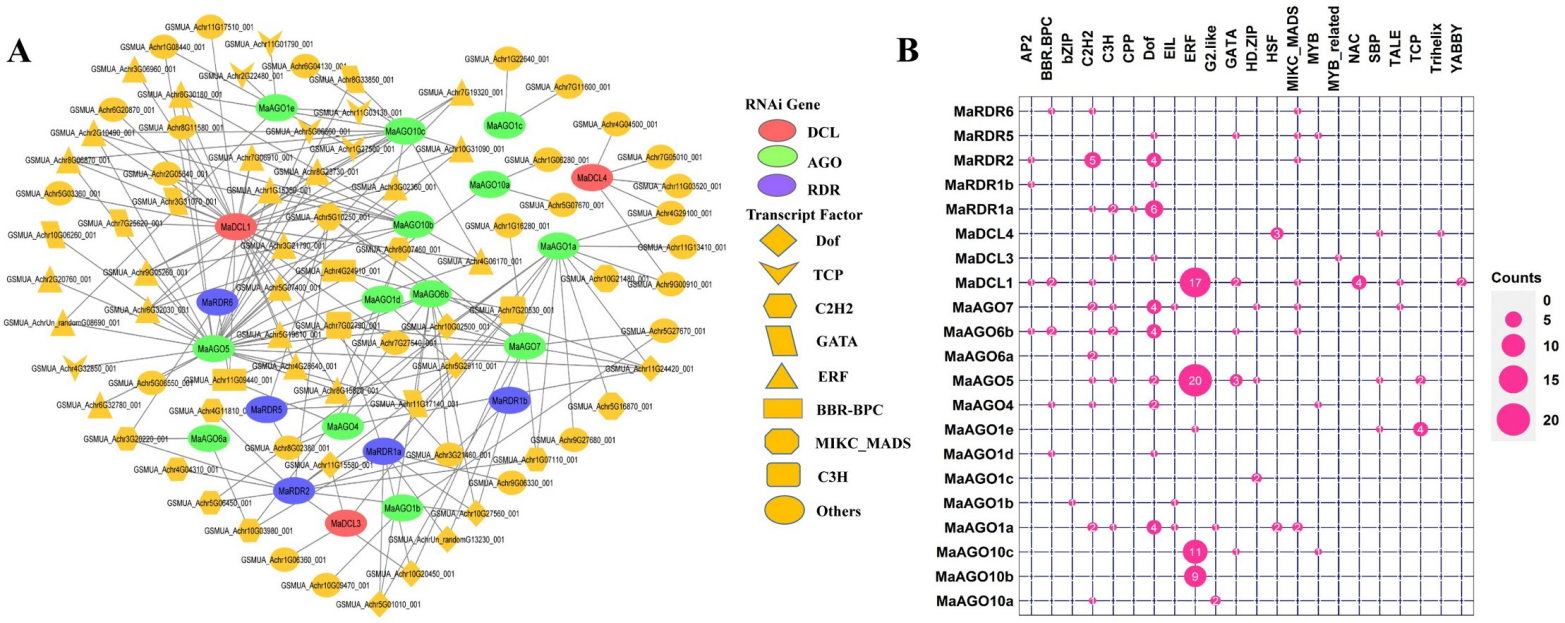
(A) The regulatory network among the TFs and the predicted RNAi genes. Each node of the network was colored based on RNAi genes and TFs. The MaDCL, MaAGO, and MaRDR genes were represented by pink, green, and blue node color, respectively, and the TFs were represented by yellow node color. Different node symbols were used for different families of TFs. TFs were configured at the hub node level using Magenta (B). The map represents the related number of TFs with the predicted MaRNAi genes.

We identified a total of 180 TFs which can regulate the candidate RNAi genes in the banana genome (S5 File). Based on the TFs families, identified TFs were divided into 22 groups. Among the TFs families, ERF, Dof, C2H2, TCP, and GATA families included 21, 8, 7, 6, and 5 TFs and calculated 54.65% of the total identified TFs (S5 File). Our analyzed results indicate that identified TFs could play significant roles in regulating RNAi genes. Based on network analysis, the identified TFs family showed a unique structure and linked to the candidate RNAi genes (Fig 12A). Likely, the ERF is dominantly associated with RNAi gene MaAGO5 (Fig 12B, S5 File). In addition, the ERF is also related to MaDCL1, MaAGO10b, and MaAGO10c, which are liked to MaAGO5 (Fig 12B).

**Fig 13:**
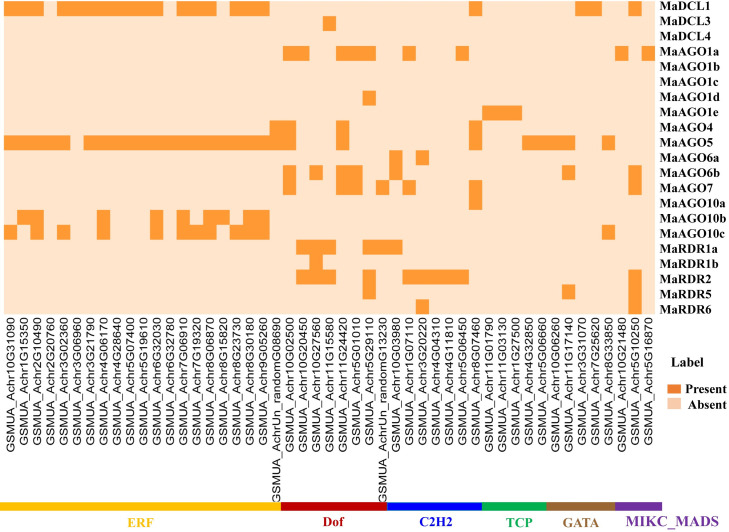
RNAi gene-mediated sub-network for ERF, Dof, C2H2, TCP, GATA and MIKC_MADS TFs families which is expressed as Heatmap.

We also analyzed the sub-network relationship between TFs and predicted MaDCL, MaAGO, and MaRDR genes (Fig 13). The sub-network analysis results revealed that TFs family ERF linked to MaDCL genes (MaDCL1), MaAGO genes (MaAGO1e, MaAGO5, MaAGO10b and MaAGO10c) except MaRDR genes. Similarly, TFs Dof family associated with MaDCL genes (MaDCL3), MaAGO genes (MaAGO1a, MaAGO1d, MaAGO4, MaAGO5, MaAGO6b and MaAGO7) and MaRDR genes (MaRDR1a, MaRDR1b, MaRDR2 and MaRDR5). However, TFs family C2H2 constructs the regulatory relationship with MaDCL genes (MaDCL1), MaAGO genes (MaAGO1a, MaAGO4, MaAGO5, MaAGO6a, MaAGO6b, MaAGO7 and MaAGO10a) and MaRDR genes (MaRDR1a, MaRDR6 and MaRDR2). Interestingly, there were only two MaAGO genes (MaAGO1e and MaAGO5) containing TFs of the TCP family. The GATA TF is associated with MaDCL genes (MaDCL1), MaAGO genes (MaAGO5, MaAGO6b and MaAGO10c) and MaRDR5 genes. By the node degree analysis, we identified five hub TFs which contained at least five associated predicted RNAi genes (Fig 13). Our identified hub TFs interacted with twelve RNAi genes. Among the twelve RNAi genes corresponding to five hub TFs, one is MaDCL, seven are MaAGO and four are MaRDR. Out of five hub TFs, 3 belonged to Dof TFs family, one is C2H2 and one is MIKC_MADS TFs family (Fig 13). Previous studies suggested that Dof TFs family is associated with the DNA-binding activities in the N-terminal and C-terminal region of target RNAi genes and they regulated the target gene expression in several plant species. The Dof TFs family is involved in i) controlling the phenylpropanoid and glucosinolates metabolism, ii) influencing the seed germination, iii) controlling stress tolerance and iv) flowering time-period [135–139]. A transcriptional repressor, MaDof23 physically interacts with MaERF9 and act as a regulator of fruit ripening which could be associated with regulation of others genes related to cell wall degradation and aroma formation in banana [140]. The MYB TFs family is found in both animals and plants. MYB TFs family in plants is considered to be the largest TFs family which contained MYB domain (a 52–53 aa residues motif) located in their N- and C-terminal region and have important role in metabolism, biotic and abiotic stress, and defense against pathogen attack [141–143]. An R2R3-MYB TF MaMYB3 showed the fruit ripening activity through the modulation of starch degradation process [144]. In various plant species, the WRKY family is believed to be involved in i) the modulation of antagonistic interaction between salicylic acid and jasmonic acids signaling and ii) the enhancement of defense against neurotropic pathogens [145,146]. Previous studies have shown that MaWRKY52 exhibited resistance against a major destructive nematode, *Pratylenchus coffeae* of banana [147]. However, it is important to note that the WRKY TFs family is also considered as anti-microbial signals molecules [148]. Prakash and Chakraborty revealed that various TFs, including MYB, WRKY, and NAC TFs could regulate the expression of RDR gene families under different stress conditions [149]. These TFs are involved mainly in plant growth, development, and response to a variety of stress conditions [148,150–152]. Plant calmodulins and calmodulin-related proteins play significant roles in regulating defense-related genes interacting with various TFs such as MYB, WRKY, and NAC [153,154]. An overexpression of *MusaNAC042* demonstrated a positive correlation related to salinity and drought resistance in *Agrobacterium*-mediated transgenic banana [155]. Another important TFs family, MIKC_MADS also involved in the transcription of several RDR genes. For example, the *OsRDR1* gene could enhance the response to the rice stripe virus (RSV) in rice (*O*. *sativa*) [156]. Two banana MADS-box family members, MaMADS24, and MaMADS49 have shown the interactions with various proteins such as hormone-response proteins, ethylene signal transduction and biosynthesis-related proteins, starch biosynthesis proteins and metabolism-related proteins associated with fruit development and ripening [157]. The regulatory network and sub-network showed that RNAi process of predicted putative genes in *M*. *acuminata* represents a diagrammatic evolutionary model that could be explored in detail through the characterization of these predicted genes. Further gene function analysis studies are required to investigate the biosynthesis pathway of calmodulins and calmodulin-related proteins, which could be involved in RNA-related pathways in *M*. *acuminata*.

### 3.9 Prediction of *cis‑*acting regulatory elements of DCL, AGO and RDR proteins in banana

**Fig 14:**
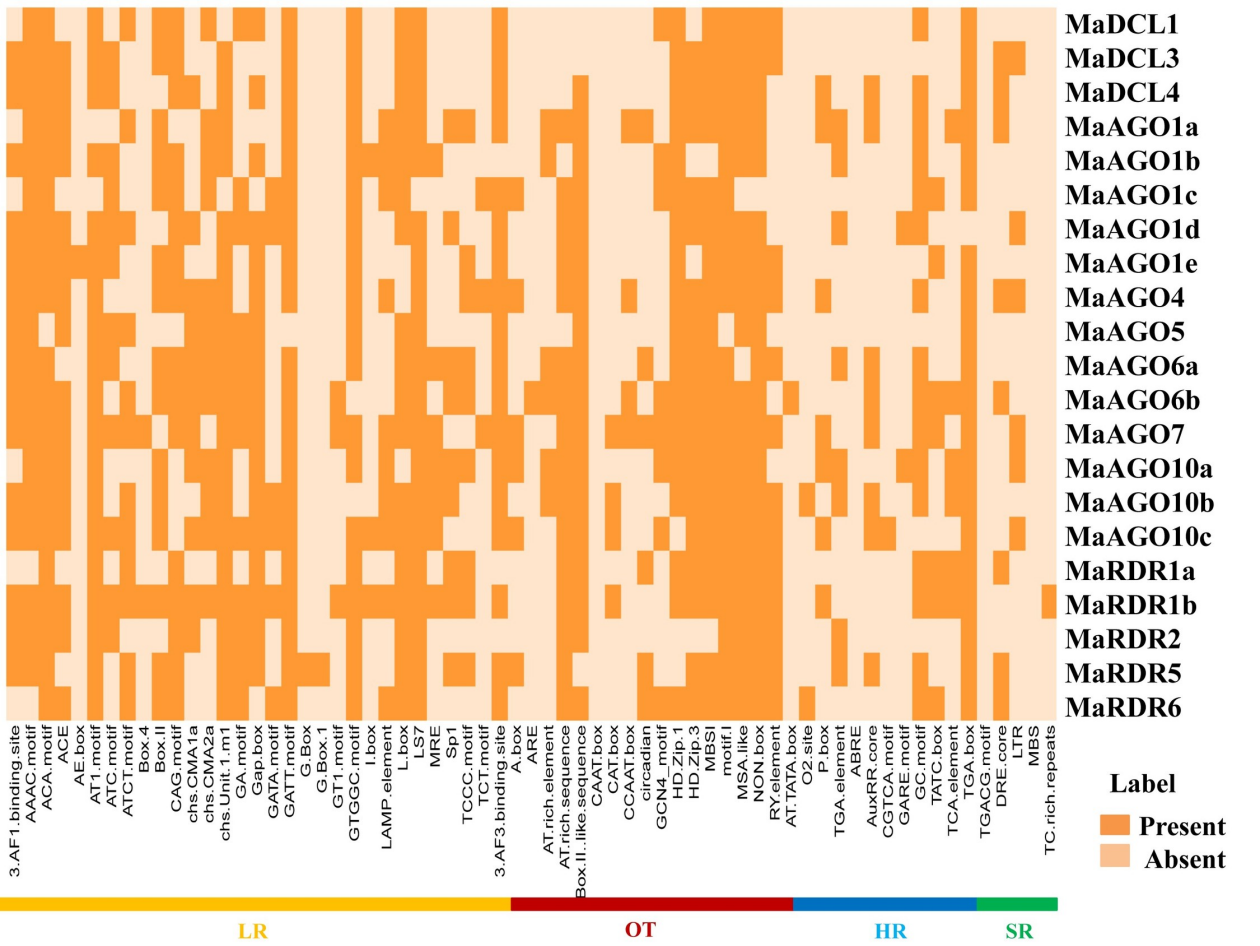
The CAREs in the upstream promoter region of predicted MaDCLs, MaAGOs and MaRDRs genes, respectively. The deep color represents the presence of that element with the corresponding genes.

The *cis*-regulatory elements (CAREs) are typically non-coding DNA composed of the short motif (5–20 bp), which is located in the promoter region of target genes [158,159]. By binding the target sites of CAREs, TFs and transcriptional regulators (up-regulator/down-regulator) control the transcriptional process and act as gene regulators [159]. Recently, a large number of sequencing data on economically important crops are being deposited each year through the advancement of high-throughput genome sequencing techniques [160]. Therefore, we can easily access the database and search these functional regulatory elements with specific gene functions within their DNA sequence (typically promoter and enhancer region) by using integrated bioinformatics techniques. We determine the various important motifs, their functional roles, and diversity of the predicted DCL, AGO, and RDR genes in banana by CAREs analysis (S6 File; Fig 14).

Analyzed results showed that LR, SR, HR, and OT-associated motifs were present in the upstream regulatory regions of RNAi genes (S6 File; Fig 14). Photosynthesis is a crucial physiological parameter and involved with the light response, which occurs in the leaves tissue of bananas [161]. Among the motifs, the LR motifs were abundant in the upstream regulatory regions of RNAi genes when compared with the other motifs. LR motifs, 3AF1 binding site, AAAC motif, ACA motif, AT1 motif, ATC-motif, ATCT-motif, box-II, chs unit1m1, GA motif, GATT-motif, GTGGC motif, LAMP element, L-box, LS7, and 3AF3 binding site were dominantly shared by the huge numbers of predicted RNAi genes in banana. Besides this, some other important LR motifs were recognized in this study, such as ACE, box-4, CAG motif, chs CMA1a, chs CMA2a, Gap box, GATA motif, G-box, G box1, GT1 motif, MRE, Sp1, TCCC motif, TCT motif which have been shared their CAREs by RNAi genes in banana. Previous studies have shown that these predicted LR-related motifs are greatly involved in the light response of different species [162–165]. An *in silico* analysis of DCL, AGO, and RDR gene families in *C*. *sinensis* also identified almost similar LR motifs, which were predicted to be involved in the leaves’ photosynthesis process [5]. Therefore, predicted motifs related to LR could play a significant role in the photosynthesis mechanism in banana leaves, which can increase grain quality and productivity.

We detected AT-rich sequence, boxII-like sequence, HD Zip1, HD Zip3, MBS1, motif1, MSA-like, Non-box, RY elements associated with various plant biological functions are highly shared by many RNAi genes predicted in banana (Fig 14). Plant hormones are also called plant growth regulators, which individually or coordinately play regulatory roles in plant growth and development activities [166–169]. These plant growth regulators have important biological functions in seed germination, plant growth, development and metabolism activities [158,170–174]. We also predicted various plant HR motif such as O_2_ site, P-box, TGA elements, Aux RR core, GC motif, TATC box, TCA elements, TGA box, which are shared by most of the identified RNAi genes in banana. The prediction of HR-related motifs suggests their important biological functions in bananas.

Moreover, DRE core, LTR, and TC-rich repeats were shared with several predicted RNAi genes. Several research groups evaluated that TC-rich repeats, LTR elements, MBS, and DRE act as SR motif in different plant species [5,175–178]. In addition, some unknown CAREs were recognized in this study (S6 File). Collectively, CAREs shared by the putative RNAi gene family in the banana will provide valuable information on their functional roles in plant growth, development, and defense against microbial infection.

## 4 Conclusion

In this study, we performed a set of integrative bioinformatics approach to *in silico* identification of RNAi pathway genes in the banana genome. In total, 3 DCL, 13 AGO, and 5 RDR RNAi pathway genes were identified in the banana genome. The phylogenetic analysis demonstrated that all subfamilies of RNAi genes maintain their maximum higher evolutionary relationship corresponding to banana and Arabidopsis RNAi genes. Conserved domain, motif, and gene structure revealed their functional similarity to those of banana and Arabidopsis RNAi genes. GO analysis revealed that most of the identified MaDCL, MaAGO, and MaRDR genes are connected with important biological functions; RNA silencing, defense against pathogens, and metabolic activity. Sub-cellular localization prediction exhibited that most of the MaDCL, MaAGO, and MaRDR proteins were abundant in the cytoplasm, and nucleus, where the PTGS process mainly occurred. Regulatory network and sub-network analysis were identified important TFs; ERF, Dof, C2H2, TCP, GATA and MIKC_MADS families, which are associated with MaDCL, MaAGO, and MaRDR genes. Also, analyzed CAREs associated with LR, SR, and HR were predicted to bind the TFs of putative MaDCL, MaAGO, and MaRDR genes. Therefore, our findings will provide valuable information on DCL, AGO, and RDR genes in the banana genome, which might be helpful for the cloning and characterization of banana RNAi genes in wet lab conditions and further improvement of these genes for the breeding programs of this economically important crop species. Moreover, these findings could upgrade the knowledge to *in silico* analysis of genes related to the various important biological pathways from others crop species.

## Supporting information

S1 File Full-length protein sequences of DCL gene families of *A*. *thaliana* and *M*. *acuminata* plant species. (TXT)

S2 File Full-length protein sequences of AGO gene families of *A*. *thaliana* and *M*. *acuminata* plant species. (TXT)

S3 File Full-length protein sequences of RDR gene families of *A*. *thaliana* and *M*. *acuminata* plant species. (TXT)

S4 File The details GO analysis of the predicted RNAi related genes was performed using online tool of Plant Transcription Factor Database (PlantTFDB, http://planttfdb.cbi.pku.edu.cn//). (XLSX)

S5 File Identified in total 180 TFs associated the regulation of predicted RNAi silencing genes in banana genome. (XLSX)

S6 File The predicted *cis*-acting regulatory elements of the upstream promoter region (1.5 kb genomic sequences) of RNAi gene families in banana. (XLSX)

## Abbreviations

RNAi: RNA interference
AGO: Argonaute
DCL: Dicer-Like
RDR: RNA-dependent RNA polymerase
miRNA: microRNA
PTGS: post transcriptional gene silencing
RISC: RNA induced silencing complex
sRNA: small RNA
dsRNA: double-stranded RNAs
mRNA: messenger RNA
siRNA: short interfering RNA
TFs: transcription factors
ORF: open reading frame
aa: amino acid
BLAST: Basic Local Alignment Search Tool
GO: Gene Ontology
GSDS: Gene Structure Display Server
HMM: Hidden Markov Model
MEME: Multiple Em for Motif Elicitation, LR, light-responsive
SR: stress-responsive
HR: hormone-responsive
OT: other activities
pI: isoelectric point
kD: kilo Daltons
bp: base pair
CARE: cis-regulatory element
ta-siRNAs: trans-acting small interfering RNA
